# Fe65-engineered neuronal exosomes encapsulating corynoxine-B ameliorate cognition and pathology of Alzheimer’s disease

**DOI:** 10.1038/s41392-023-01657-4

**Published:** 2023-10-23

**Authors:** Ashok Iyaswamy, Abhimanyu Thakur, Xin-Jie Guan, Senthilkumar Krishnamoorthi, Tsz Yan Fung, Kejia Lu, Isha Gaurav, Zhijun Yang, Cheng-Fu Su, Kwok-Fai Lau, Kui Zhang, Roy Chun-Laam Ng, Qizhou Lian, King-Ho Cheung, Keqiang Ye, Huanhuan Joyce Chen, Min Li

**Affiliations:** 1 Mr. & Mrs. Ko Chi-Ming Centre for Parkinson’s Disease Research, School of Chinese Medicine, Hong Kong Baptist University, Hong Kong SAR, China; 2 Pritzker School of Molecular Engineering, The University of Chicago, Chicago, IL USA; 3 Ben May Department for Cancer Research, The University of Chicago, Chicago, IL USA; 4 School of Chinese Medicine, Hong Kong Baptist University, Hong Kong SAR, China; 5 School of Life Sciences, The Chinese University of Hong Kong, Hong Kong SAR, China; 6 Division of Neuroscience, School of Biological Sciences, Faculty of Biology, Medicine and Health, Manchester Academic Health Science Centre, University of Manchester, Manchester, United Kingdom; 7 Department of Medicine, Li Ka Shing Faculty of Medicine, The University of Hong Kong, Pok Fu Lam, Hong Kong SAR, China; 8 Prenatal Diagnostic Center and Cord Blood Bank, Guangzhou Women and Children’s Medical Center, Guangzhou Medical University, Guangzhou, China; 9 HKUMed Laboratory of Cellular Therapeutics, the University of Hong Kong, Pok Fu Lam, Hong Kong SAR, China; 10 Faculty of Life and Health Sciences, Shenzhen Institutes of Advanced Technology, Chinese Academy of Sciences, Shenzhen, China

**Keywords:** Biotechnology, Neurological disorders

## Abstract

Alzheimer’s disease (AD) is a neurodegenerative disorder characterized by the predominant impairment of neurons in the hippocampus and the formation of amyloid plaques, hyperphosphorylated tau protein, and neurofibrillary tangles in the brain. The overexpression of amyloid-β precursor protein (APP) in an AD brain results in the binding of APP intracellular domain (AICD) to Fe65 protein via the C-terminal Fe65-PTB2 interaction, which then triggers the secretion of amyloid-β and the consequent pathogenesis of AD. Apparently, targeting the interaction between APP and Fe65 can offer a promising therapeutic approach for AD. Recently, exosome, a type of extracellular vesicle with diameter around 30–200 nm, has gained much attention as a potential delivery tool for brain diseases, including AD, due to their ability to cross the blood–brain barrier, their efficient uptake by autologous cells, and their ability to be surface-modified with target-specific receptor ligands. Here, the engineering of hippocampus neuron cell-derived exosomes to overexpress Fe65, enabled the development of a novel exosome-based targeted drug delivery system, which carried Corynoxine-B (Cory-B, an autophagy inducer) to the APP overexpressed-neuron cells in the brain of AD mice. The Fe65-engineered HT22 hippocampus neuron cell-derived exosomes (Fe65-EXO) loaded with Cory-B (Fe65-EXO-Cory-B) hijacked the signaling and blocked the natural interaction between Fe65 and APP, enabling APP-targeted delivery of Cory-B. Notably, Fe65-EXO-Cory-B induced autophagy in APP-expressing neuronal cells, leading to amelioration of the cognitive decline and pathogenesis in AD mice, demonstrating the potential of Fe65-EXO-Cory-B as an effective therapeutic intervention for AD.

## Introduction

There is a growing health crisis owing to the needs of dementia patients that warrants immediate attention. Currently, it is anticipated that globally, beyond 40 million people are suffering from Alzheimer’s disease (AD).^1^ With a continuously aging population, the incident rate of AD is anticipated to rise substantially.^2^ AD is a progressive disorder caused by neuron impairment in the hippocampus, accumulation of amyloid plaques, hyperphosphorylated tau protein, and neurofibrillary tangles (NFTs). AD characteristically effects or destroys neurons in the various regions of the brain associated with memory, including the hippocampus and entorhinal cortex. Subsequently, it affects parts of the cerebral cortex associated with social behavior, reasoning, and language processing.^3,4^ Major pathological elements underlie the progression of AD, namely amyloid plaques, hyperphosphorylation of tau protein, and development of NFTs.^5^ Available therapeutic options for AD that include acetylcholinesterase inhibitors, N-methyl-D-aspartate receptor (NMDA) antagonist (memantine), secretase inhibitors, amyloid binders, and inhibitors of the phosphorylation of tau proteins focus only on amelioration of symptoms or slowing the progression of damage.^6,7^ These drugs are limited in their ability to affect AD pathogenesis and progression with a consequent demand for more effective and alternative treatments.

For a decade, traditional medicine has attracted much attention in the treatment of AD due to its effectiveness in enhancing cognitive effect.^8^ Recently, Corynoxine-B (Cory-B), a natural autophagy inducer from the medicinal plant *Uncaria rhynchophylla*, has been found to be effective in AD^9^ as well as Parkinson’s disease (PD).^10^ Corynoxine isomers including Cory-B can decrease the level of amyloid β peptide and amyloid-β precursor protein (APP) by inducing autophagy in neuronal cells.^9^ Nonetheless poor brain permeability to Corynoxine isomers results in less brain bioavailability,^11^ suggesting poor access through the blood–brain barrier (BBB). Therefore, there is a need to develop a novel drug delivery system (NDDS) with a specific target in the AD brain.

Naturally occurring nanovesicles such as exosomes may serve as an effective delivery vehicle. Exosomes are extracellular vesicles (EVs) with diameter ranging around 30–200 nm that are secreted by most cell types in their extracellular milieu, and found in biofluids including blood and cerebrospinal fluid.^12–15^ Importantly, an exosome-based NDDS would have crucial benefits including biocompatibility, innate stability, low immunogenicity, and tunability of its surface with consequent targeting ability.^16,17^ In addition, exosomes can cross the BBB,^12,18^ and of note, autologous exosomes are better internalized by recipient cells, which is often referred as homing effect.^19^ Therefore, autologous exosomes can be genetically modified to carry ligands specific to receptors and deliver cargo with great stability.^20^ In this system, Fe65-engineered exosomes produced by hippocampal neurons were used to deliver Cory-B,^21^ to the brain of AD mouse models. This exosome-based targeted delivery system could provide a promising approach for treating AD.

Amyloid precursor protein (APP) a highly expressed transmembrane protein in the AD brain, and a neuronal adapter Fe65 binds to APP intracellular domain (AICD), after the proteolytic cleavage of APP by β-secretase and γ-secretase.^22^ The Fe65/APP complex is then translocated to the nucleus to initiate gene transcription, the exact function of which is still unclear. However, a recent study suggests that Fe65 may regulate transcription independent of AICD and has been implicated in long-term memory formation.^23^ Additionally, the expression of Fe65 in APP transgenic mice has shown decline in amyloid beta (Aβ) load,^20,24^ suggesting that targeting the interaction of Fe65/APP may provide a viable approach for treating AD. As Fe65 is one of the best binding partners of APP,^22^ during the APP processing in AD, APP can be cleaved by α-secretase within the Aβ domain to release soluble APP.^25^ The portion of Fe65 expressed on exosome membrane would potentially facilitate the engineered exosomes to target the region, where soluble APP is accumulated, followed by fusion of exosomes to the neuronal cells to release its content via hemi-fusion stalk formation between hydrophobic lipid bilayers of the exosome and plasma membrane.^26^ To this end, a novel APP-targeted brain drug delivery system based on exosomes has been developed.

Further, the progression of AD has long been associated with damage to the autophagy-lysosomal degradation pathway via Beclin-1 (BECN1), which plays a crucial role in the lipidation of LC3B-I to LC3B-II and induces autophagy flux. Interestingly, BECN1 has been found to play role in internalization of membrane APP via their sorting for lysosomal degradation. The activation of BECN1 initiates the autophagy pathway by recruiting ATG5 and ATG7 to the plasma membrane. The autophagosome, formed with the assistance of these proteins, envelops cellular materials such as damaged organelles and misfolded proteins. The autophagosome is then moved to the lysosome, where it merges with the lysosome membrane to break down the contents of the autophagosome.^27–29^ Therefore, we examined whether Fe65-EXO-Cory-B could enter neuron cells through APP receptor-dependent endocytosis and induce autophagy via BECN1, ATG5, and ATG7 to reduce AD pathology and improve cognitive and locomotor behavior. Therefore, Fe65-EXO-Cory-B as a potentially effective targeted AD therapy may serve as an intervention to ameliorate AD pathogenesis.

## Results

### Development and characterization of Fe65-engineered hippocampus neuron cell-derived exosomes

**Fig. 1:**
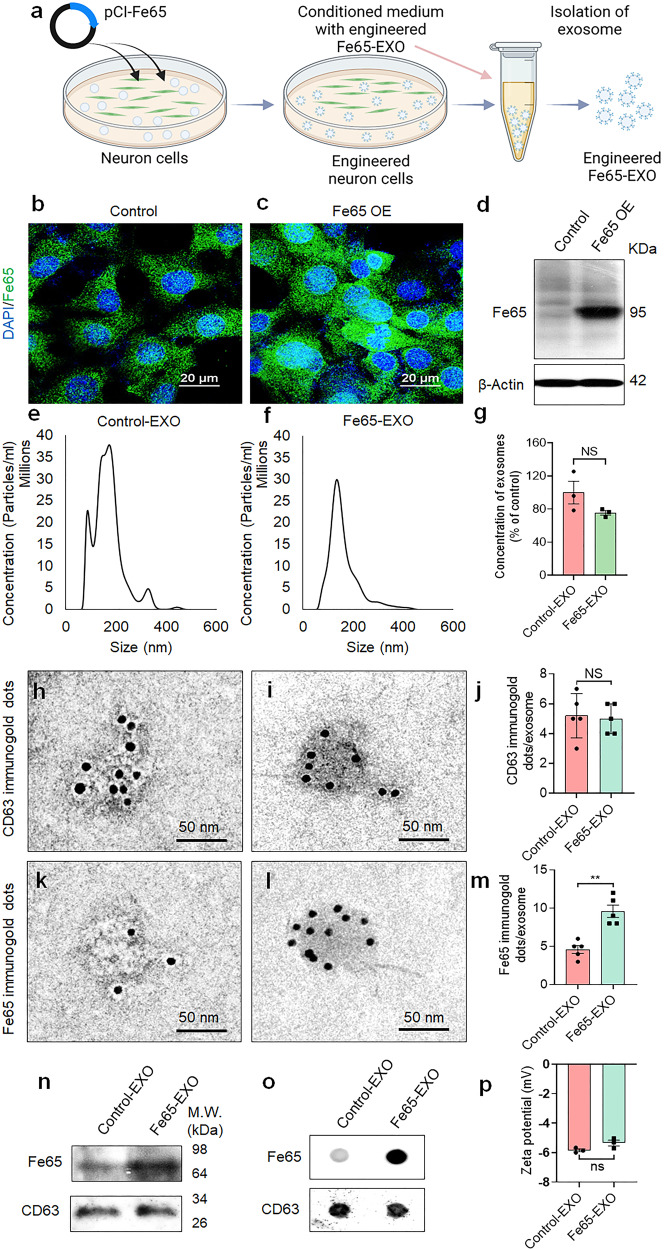
Development and characterization of Fe65-engineered exosomes. **a** Schematic diagram showing the steps involved in the production of engineered Fe65-EXO. Representative **b**, **c** immunofluorescence images showing expression of Fe65, **d** protein level of Fe65 in control- and Fe65 OE- HT22 hippocampus neuron cells. Representative **e**–**g** size distribution, **h**–**j**, **k**–**m** CD63-, and Fe65-positive immunogold dots, and their relative quantification, **n**, **o** protein level of Fe65 by **n** WB, and **o** dot-blot, and **p** zeta potential of exosomes isolated from control- and Fe65 OE- HT22 hippocampus neuron cells. In **g**, **j**, **m**, and **p** data are shown as mean ± standard error mean (SEM). Statistical analysis was performed by Student’s *t*-test to compare Control-EXO vs. Fe65-EXO. Significance level: ^**^P < 0.01, NS not significant

In the brain, the hippocampus is crucial for learning and memory and is particularly vulnerable at an early stage of AD progression. There is evidence that altered neurogenesis in the hippocampus symbolizes an early key event in AD.^30^ The homing capacity of autologous exosomes makes them suitable as a drug delivery vehicle due to their better internalization by recipient cells.^19,31,32^ We utilized HT22 mouse hippocampus neuron cell-derived exosomes (EXO). As noted before, APP, which is involved in the AD pathogenesis, collaborates with its binding protein Fe65 via interaction between C-terminal Fe65-PTB2 and AICD.^33^ We engineered EXO via overexpression (OE) of Fe65 in HT22 cells by transfecting pCI-Fe65 plasmid. Immunofluorescence microscopy and western blot analysis of pCI-Fe65myc plasmid transfected HT22 cells confirmed the enhanced expression of Fe65 relative to the control group (Fig. 1a–d). Next, exosomes were isolated from the control- and Fe65 OE- HT22 cells and their size distribution, morphology, and expression of CD63 (exosomal marker)-, and Fe65- immunogold dots characterized. Nano-tracking analyzer (NTA) showed that the average size distribution of exosomes isolated from untreated HT22 cells (control-EXO; control exosomes) and Fe65 OE-HT22 cells (Fe65-EXO; engineered exosomes) was predominantly around 180 nm in diameter. Nonetheless, after Fe65 OE in HT22 cells, the number of Fe65-EXO was slightly decreased (Fig. 1e–g), suggesting that the genetic engineering of the source cells, HT22, had no significant effect on the release of exosomes. Further, the presence of CD63 positive immunogold dots in control-EXO and Fe65-EXO and their quantification (Fig. 1h–j), confirmed the presence of exosomal biomarker, with insignificant difference in the engineered exosomes as compared to the control exosomes. Notably, the significantly enhanced number of Fe65-positive immunogold dots in the engineered exosomes compared with control exosomes confirmed the effective engineering of EXO via Fe65 (Fig. 1k–m). In addition, the morphology of exosomes was intact after engineering of their surface with Fe65, with most exosomes’ circular shape (Fig. 1h, i, k, l). Further quantitative analysis via WB and dot-blot assay confirmed that the level of Fe65 was enhanced in engineered exosomes compared with control exosomes although the level of CD63 remained similar in both (Fig. 1n, o). The slightly altered zeta potential and membrane property showed that the surface charge of exosomes was not affected by the engineering process (Fig. 1p, Supplementary Fig. S1a–c), suggesting the successful and efficient engineering of neuronal exosomes with little effect on their biophysical properties.

### Fe65-engineered exosomes are efficiently internalized by APP-overexpressed neuronal cells

**Fig. 2:**
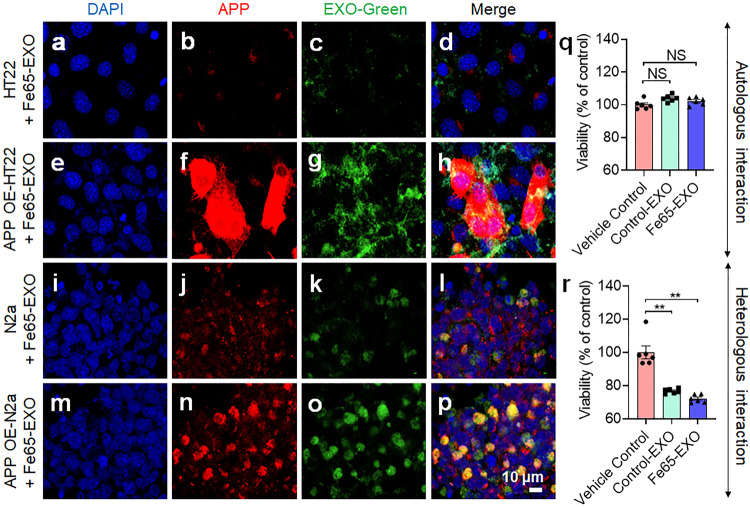
Higher autologous uptake of Fe65-engineered exosomes by APP overexpressed hippocampus neuron cells. **a**–**h** Enhanced autologous uptake of Fe65-EXO by APP OE-HT22 cells compared with HT22 cells, and **i**–**p** enhanced heterologous uptake of Fe65-EXO by APP OE-N2a cells compared with N2a cells after 24 h. Nuclei: DAPI (blue), cell membrane: APP (red), Fe65-EXO: Green (green). EXO-APP (yellow). In **a**–**p**; scale bar = 10 µm. **q**, **r** Effect of Fe65-EXO on the viability of HT22- and N2a- neuron cells, respectively. HT22- and N2a- neuron cells were treated with cell culture medium (control), control EXO, or Fe65-EXO for 24 h, followed by the measurement of viability using MTT assay protocol. In **q** and **r**, data are shown as mean ± S.E.M (*N* = 6). Statistical analysis was performed by one-way ANOVA for multiple comparison of Vehicle Control vs. Control EXO or Fe65-EXO, Significance level: ^**^P < 0.01, NS not significant

As noted previously, Fe65 is a brain-enriched phospho-adapter protein that interacts with APP to regulate its processing.^22,34^ We examined the ability of Fe65-EXO to be internalized by APP over-expressing neuron cells. To understand whether Fe65 engineering of exosomes could enhance the uptake of Fe65-EXO in the APP-targeted neuron cells, we examined the dose dependent effect of control-EXO on the viability of HT22 cells (Supplementary Fig. S1d), followed by evaluating the effect of Fe65-EXO on APP over-expressing neuron cells. OE of APP was performed in the HT22 and N2a cells by transfecting with pCAX APP Swe/Ind (Plasmid# 30145, Addgene). Next, Exo-Green labeled Fe65-EXO were added to the medium containing culture of control HT22, APP OE-HT22, control N2a, and APP OE-N2a cells (Fig. 2a–r). Notably, the uptake of Fe65-engineered exosomes was augmented by both APP OE-HT22 and APP OE-N2a cells (Fig. 2e–h, m–p) compared with their respective control groups (Fig. 2a–d, i–l). This suggests that Fe65 engineering enhanced the delivery of exosomes to the APP OE neuron cells, signifying their potential application for APP-targeted therapeutic delivery in AD.

To ascertain the effect of control-EXO and Fe65-EXO on the viability of neuron cells, HT22- and N2a- cells were treated with cell culture medium (control), control-EXO, or Fe65-EXO in a 96-well plate, followed by the measurement of viability after 4 h-incubation (Fig. 2q, r). The result showed that treatment of control-EXO or Fe65-EXO did not inhibit proliferation of autologous HT22 cells, whereas control-EXO or Fe65-EXO reduced the proliferation of heterologous N2a cells (Fig. 2q, r), suggesting that autologous control-EXO or Fe65-EXO may be a suitable carrier to deliver AD therapeutics.

### Fe65-engineered exosomes encapsulating Cory-B enhanced autophagy in neuronal cells

**Fig. 3:**
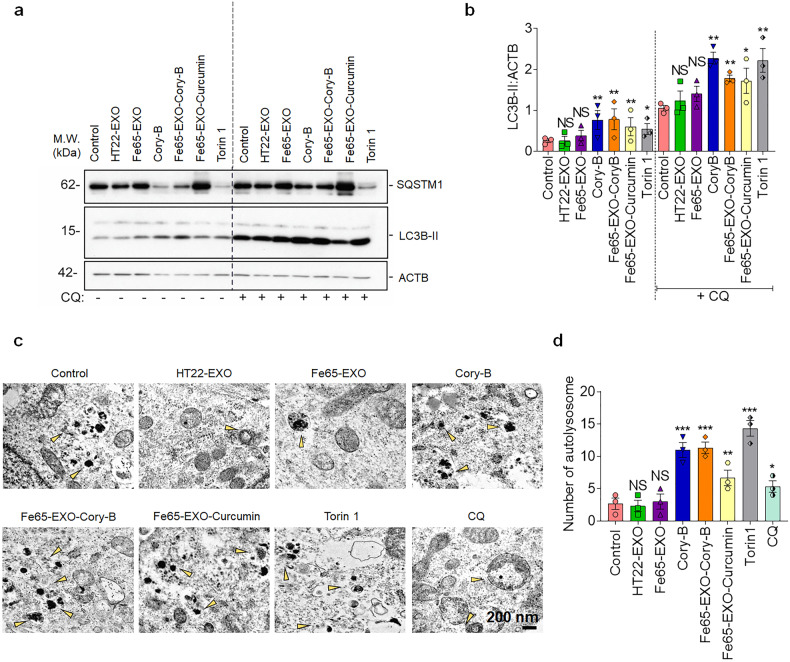
Delivery of Cory-B by Fe65-EXO caused BECN1-dependent augmented autophagy in neuronal cells. **a, b** Representative immunoblots depicting the protein level of autophagy markers (SQSTM1 and LC3B-II) in N2a cells, without and with pre-treatment of chloroquine (CQ, 100 μM), and the corresponding bar graphs. **c, d** Representative electron micrograph of N2a cells showing the formation of autolysosome and the corresponding bar graphs, respectively. Scale bar: 200 nm. In **b** and **d**, data are presented as mean ± SEM (*N* = 3). Statistical analysis was performed by one-way ANOVA with Dunnett’s multiple comparison test to compare Control vs. treatment groups. Significance level at ^*^P < 0.05, ^**^P < 0.01, and ^***^P < 0.001

Damage to the autophagy-lysosomal degradation pathway has been linked with AD pathogenesis.^28,29^ Microtubule Associated Protein 1 Light Chain 3 Beta (MAP1LC3B) (or LC3B-I), a commonly used autophagy marker is localized in the cytosol. After activation of autophagy, LC3B-I is lipidated into LC3B-II and has been found to be involved with autophagosome membranes.^27^ Another autophagy marker, SQSTM1/p62, which is an adapter protein that connects LC3B and the aggregation of ubiquitinated protein leads to autophagy degradation.^35^ Since Cory-B is a natural autophagy inducer, it can be hypothesized that the delivery of Fe65-EXO-Cory-B will further enhance autophagy.^21^ The Cory-B was loaded into Fe65-EXO via sonication with drug loading efficiency of approximately 28% (Supplementary Fig. S2a, b). To further validate whether Fe65-EXO-Cory-B could promote autophagy flux, N2a neuronal cells were treated with Fe65-EXO-Cory-B either with or without lysosomal inhibitor, chloroquine (CQ, 100 μM) that has been widely utilized to detect autophagy flux.^36^ Importantly, treatment of Fe65-EXO-Cory-B in the absence or presence of CQ, enhanced the accumulation of LC3B-II in N2a cells (Fig. 3a, b). Immunofluorescence imaging showed significantly enhanced formation of green puncta of GFP-LC3B-II in N2a cells treated with Fe65-EXO-Cory-B (Supplementary Fig. S3a, b). Additionally, augmented red puncta (depicting autolysosomes) was observed in N2a cells treated with Fe65-EXO-Cory-B that were transfected with mRFP-GFP-LC3 plasmids (Supplementary Fig. S3c, d). Electron microscopy analysis demonstrated the formation of many autolysosome structures in N2a cells treated with Fe65-EXO-Cory-B (Fig. 3c, d). Conclusively, Fe65-EXO-Cory-B can enhance autophagy in N2a cells and may be attributed to the efficient delivery of Cory-B in N2a neuronal cells by Fe65-EXO.

PIK3C3 complex comprising BECN1, plays a crucial role in the lipidation of LC3B-I to LC3B-II, and induces autophagy flux, and involves ATG5 and ATG7.^27–29^ Therefore, it was examined whether Fe65-EXO-Cory-B could induce autophagy via BECN1, ATG, and ATG7. We utilized 3-MA, a PIK3C3 complex inhibitor that can inhibit the lipidation of LC3B-I to LC3B-II.^37^ Notably, following Fe65-EXO-Cory-B treatment, LC3B-II levels were increased in neuronal cells, although 3-MA inhibited the induction of LC3B-II levels and blocked the decrease of SQSTM1 protein in Fe65-EXO-Cory-B treated neuronal cells (Supplementary Fig. S3e, f). Furthermore, the protein level of LC3B-II was significantly increased in N2a neuronal cells treated with Fe65-EXO-Cory-B and reduced upon knockdown of BECN1, ATG5, or ATG7 (Supplementary Figs. S3g, h, S4a–d), demonstrating the role of Fe65-EXO-Cory-B in stimulating autophagy in neuronal cells via either BECN1, ATG5, or ATG7.

### Fe65-EXO effectively delivered Cory-B to APP-expressing neuron cells in the AD mice brain

**Fig. 4:**
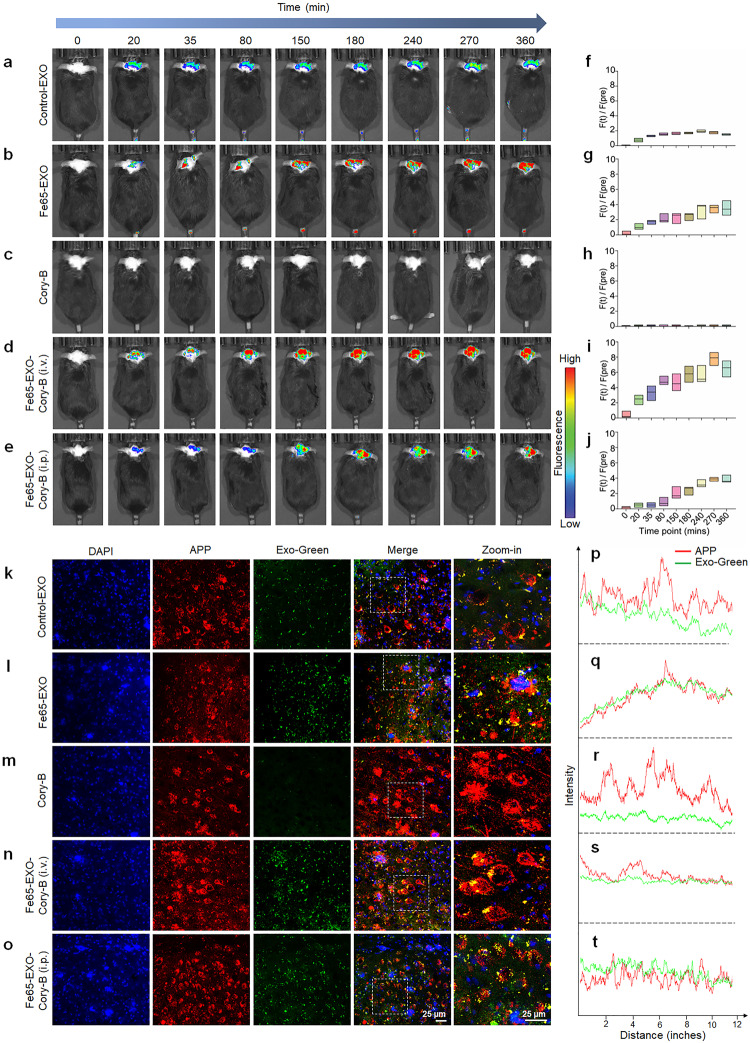
Biodistribution of Fe65-EXO-Cory-B in the brain of AD mouse. Intravenously injected **a** control exosomes, **b** Fe65-EXO, **c** Cory-B, and **d** Fe65-EXO-Cory-B in 5xFAD mouse model. **e** Intraperitoneally injected Fe65-EXO-Cory-B in 5xFAD mouse model. The exosomes are labeled with Exo-Glow dye, and after its administration, AD mice were imaged at various times using the IVIS In Vivo Imaging System. **f**–**j** The quantitative time course bar graphs show the biodistribution of control-EXO, Fe65-EXO, Cory-B, and Fe65-EXO-Cory-B (i.v. or i.p.) in the brain of a 5xFAD mouse model. **k**–**o** Immunofluorescence staining showing colocalization of APP (shown in red) and exosomes (labeled with Exo-Green; shown in green) in the hippocampus region of brain of 5xFAD mouse model treated with control-EXO, Fe65-EXO, Cory-B, and Fe65-EXO-Cory-B (i.v. or i.p.), and **p**–**t** the corresponding fluorescence intensity of APP and Exo-Green, depicting the strength of their colocalization. In **k**–**o**, scale bar = 25 µm

Effective treatment of AD requires therapeutic agents that can cross the BBB and be successfully delivered to the brain. To examine the ability of Fe65-EXO to enable the delivery of Cory-B to the brain, Exo-Glow labeled control-EXO, Fe65-EXO carrying Cory-B were intravenously administered to the 5xFAD mouse. In addition, Cory-B alone was injected intravenously, and Fe65-EXO-Cory-B was injected in another group of 5xFAD mice intraperitoneally. After injection, the time-dependent fluorescence level of EXO-Glow labeled exosomes were monitored using the IVIS bioluminescence in vivo imaging system. Importantly, approximately 30 min after injection, the Exo-Glow labeled Fe65-EXO showed higher fluorescence in the 5xFAD mouse brain than the control exosomes, and continued to increase in a time-dependent manner (Fig. 4a, b) This suggested that Fe65 engineering augmented the capacity of exosomes to cross the BBB. Also, intravenous Cory-B and Cory-B carrying-Fe65-engineered exosomes injected intravenously and intraperitoneally were also tracked (Fig. 4c–e). Interestingly, the Fe65-EXO-Cory-B administered intravenously showed higher fluorescence in the AD mouse brain compared with those administered intraperitoneally (Fig. 4d, e). This showed that that Fe65-EXO could serve as an ideal vehicle to transport BBB-impermeable drugs including Cory-B. After tracking the fluorescence of the Exo-Glow labeled exosomes administered to the AD mice for 6 h, the mice were sacrificed. Next, heart, lung, kidney, brain, liver, and spleen of AD mice were harvested to examine the entry of control exosomes, or Fe65-EXO, or Fe65-EXO-Cory-B by measuring the fluorescent label of Exo-Glow labeled exosomes. Although the heart, lung, kidney, and liver showed accumulation of exosomal fluorescence, the brain showed significantly high exosomal fluorescence, indicating a higher affinity of Fe65-EXO for the brain. Nevertheless, no accumulation of control exosomes or Fe65-EXO was found in the spleen (Supplementary Fig. S5a–e).

Although Fe65-EXO accrued in the brain is significantly high amount as compared to other organs of AD mice (Fig. 4a–j), it was crucial to understand whether Fe65-EXO were specifically interacting with APP in the brain of AD mice. Therefore, we examined the co-expression of APP and Exo-Glow labeled- control-EXO, or Fe65-EXO, or Fe65-EXO-Cory-B (Fig. 4k–t). It was evident that Exo-Glow labeled- Fe65-EXO, or Fe65-EXO-Cory-B showed higher co-expression with APP compared with control-EXO labeled with Exo-Glow (Fig. 4a–j, k–t). Conclusively, the Fe65-EXO were able to cross the BBB and facilitate delivery of Cory-B to the brain of AD mice.

In our therapeutic study, we found that astrocyte and microglia exhibited feeble uptake of Fe65-EXO or Fe65-EXO-Cory-B (Supplementary Fig. S6a–l); however, both were found to be recruited to Aβ plaques, resulting into reduced level of Aβ, TNFɑ, and IL-1β in 5xFAD mice brain (Supplementary Fig. S7a–i). The enhanced protein level of LC3B-II and reduced SQSTM1 in the brain of 5xFAD mice injected with Fe65-EXO-Cory-B established its ability to augment autophagy, leading to the decreased level of APP-CTF (Supplementary Fig. S8a–d), Aβ and amyloid plaque in the brain of 5xFAD mice (Supplementary Figs. S9a–d, S10a–d). As the reduction of neurogenesis in AD contributes to cognitive decline,^38,39^ it is essential to investigate whether Fe65-EXO-Cory-B can enhance neurogenesis. Notably, Fe65-EXO-Cory-B has been observed to stimulate neuroprotection (Supplementary Figs. S11a–e, 12a, b).

To further verify the ability of Fe65-EXO to enhance the bioavailability of Cory-B in the brain of AD mice, the amount of Cory-B was examined in the brain relative to that in the plasma by liquid chromatography-mass spectroscopy (LC–MS) analysis of biofluid from the brain and plasma (Supplementary Fig. S13a–f). Interestingly, the amount of Cory-B was augmented in the brain by use of Fe65-EXO as a delivery carrier when administered intravenously. Moreover, the amount of Cory-B was augmented in brain biofluid relative to that in the plasma when Fe65-EXO were used as an intravenously administered delivery vehicle (Supplementary Fig. S13a–f). Importantly, there was no toxicity to any of the major organs, including the brain of AD mice who received Fe65-EXO-Cory-B (Supplementary Figs. S14–16).

### Cory-B loaded Fe65-engineered exosomes improved cognitive and locomotor behavior in AD mice

**Fig. 5:**
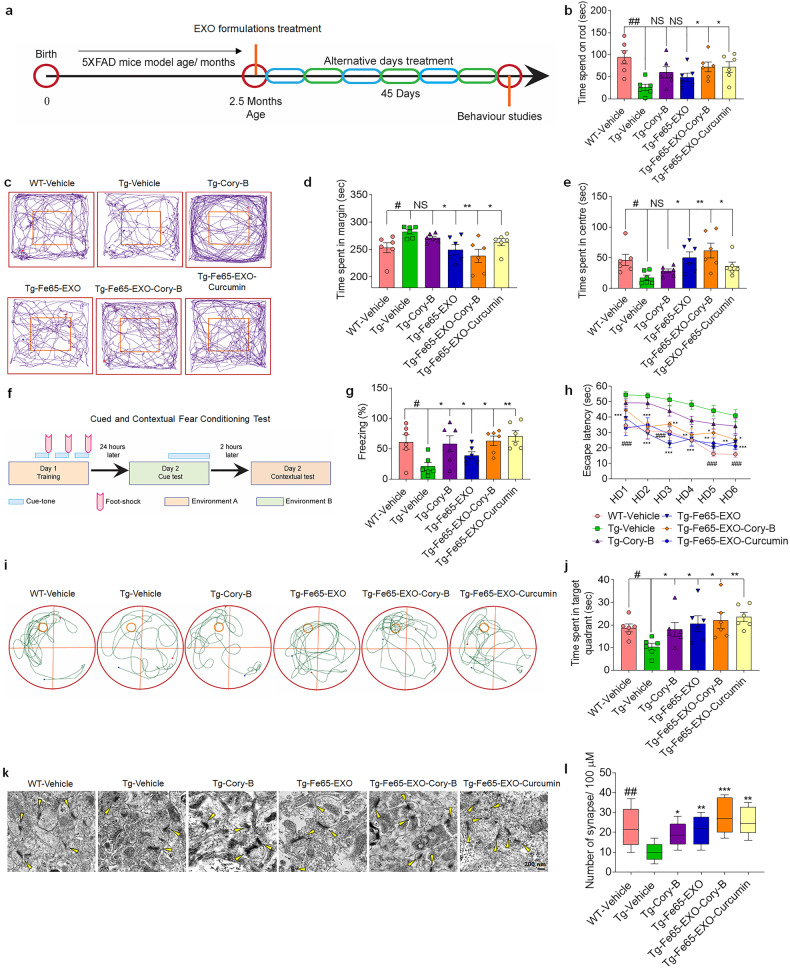
Fe65-EXO-Cory-B improves cognitive behavior in AD mice. **a** A schematic plan of the experiment showing the treatment schedule, followed by behavior experiments in 5xFAD mice. **b** Representative bar graph shows the time spent by the AD mice on the accelerating rotarod platform under various treatment conditions, as analyzed by rotarod test. **c**–**e** Analysis of general activity level, gross locomotor activity, and exploration habits of AD mice by open field test in a white Plexiglas box under various treatment conditions. Bar graphs **d** and **e** show the time spent by the AD mice in the margin and center of the Plexiglas box, based on the recorded tracks of the mice as shown in **c**. **f** Diagrammatic illustration of cued and contextual fear conditioning. **g** Representative graph showing freezing time of AD mice under various treatment conditions after the cue tone, as analyzed from the contextual fear conditioning. **h** Representative graph showing the escape latency of AD mice. **i, j** Analysis of spatial learning and memory in AD mice by Morris’s water maize (MWM) test under various treatment conditions. Representative **i** graph showing time spent by AD mice in target quadrant based on their swim paths or mouse trajectory **j** recorded in MWM. **k, l** Representative **k** electron microscopy images showing the formation of synapses (scale bar: 200 nm), and **l** the corresponding bar graph depicting the number of synapses formed in the hippocampus region of AD mouse brain under various treatment conditions. Data are presented as mean ± SEM (*N* = 6 mice/group). Statistical analysis was performed by one-way ANOVA with Dunnett’s multiple comparison test to compare Tg-Vehicle vs. treatment groups. Significance level at ^*^P < 0.05, ^**^P < 0.01, ^***^P < 0.001. Comparison of Tg-Vehicle vs. WT-Vehicle group. Significance level at ^#^*P* < 0.05, ^##^*P* < 0.01, ^###^*P* < 0.001

Aggregation of Aβ has been associated with memory deficiency, synaptic dysfunction, loss of plasticity in neurons, and neurodegeneration in AD.^40,41^ To determine whether Fe65-EXO-Cory-B could improve spatial learning and memory, various behavior tests including the rotarod test, open field test, contextual fear conditioning (CFC) test, and Morris’s water maize (MWM) test were conducted in 5xFAD mice that received Cory-B, Fe65-EXO, Fe65-EXO-Cory-B or Fe65-EXO-Curcumin, according to the experimental plan as shown in Fig. 5a.

The motor coordination of 5xFAD mice with various treatments was analyzed by rotarod test, where mice were placed on a horizontal rod that rotated about its long axis (Day-1 at 4 rpm, Day-2 at 8 rpm and Day-3 at 12 rpm and Final day at 4–40 rpm for 300 s, accelerating version), and were required to walk forwards to maintain an upright position and avoid falling off. Notably, 5xFAD mice that received Fe65-EXO-Cory-B, could spend significantly more time on the rod than the vehicle- or Cory-B treated AD mice (Fig. 5b), suggesting a role of Fe65-EXO-Cory-B in recovering AD associated motor coordination defects. Next, general activity level, gross locomotor activity, and exploratory behavior were examined in 5xFAD mice with various treatments using an open field test in a white Plexiglass box. Mice were positioned in the center of a novel environment in an open field and permitted to roam freely for around 5 min with simultaneous video recording by an overhead camera. The time spent by the 5xFAD mice in the center and margins was determined based on their trajectory in a pre-defined zone of the open field using ANY-maze software (Stoelting Co.). Interestingly, the 5xFAD mice treated with Fe65-EXO-Cory-B spent significantly longer in the center of the open field (Fig. 5c–e), suggesting that Fe65-EXO-Cory-B could effectively recover gross locomotor activity and exploratory defects in AD mice. Next, the hippocampal-associated learning ability and memory were examined by recording the freezing time in 5xFAD mice with various treatments by simple cues and/or learning by complex stimuli (context). The mice were trained with a cue tone followed by a foot shock training in day 1 and followed by cue tone assessments in day 2 (Fig. 5f). Importantly, Fe65-EXO-Cory-B treated 5xFAD mice showed an augmented cue tone-associated freezing time (Fig. 5g), suggesting the potential role of Fe65-EXO-Cory-B in improving hippocampal-associated learning ability and memory. Next, spatial learning and memory were analyzed in 5xFAD mice with various treatments applying the MWM test and by tracking their swimming trajectory. The swimming ability and vision capacity of the 5xFAD mice were evaluated by subjecting them to visual training for one day. Followed by the next 6 days hidden platform training was applied for training the 5xFAD mice and the study animals. The platform was removed on the 7th day with the mice ready to probe the platform by utilizing the animal tracking ANY-maze software to test their memory. Interestingly, the vehicle-treated 5xFAD mice exhibited slower learning and a long escape latency compared with the WT mice during learning sessions, indicating the existence of learning deficits in the vehicle group. Notably, Fe65-EXO-Cory-B treatment showed its function in rescuing the learning impairment in 5xFAD mice that showed a shorter latency to escape. After the hidden platform test, the AD mice were subjected to the memory recovery test in a probe trial. Results showed that 5xFAD mice treated with Fe65-EXO-Cory-B probed the platform more slowly in the target quadrant than those treated with vehicle. In conclusion, the results of the MWM test demonstrated that Fe65-EXO-Cory-B helped to rescue the learning and memory impairment in 5xFAD mice (Fig. 5h–j). This was also supported by the result of novel object recognition index (Supplementary Fig. S17a, b).

The potential cause of memory deficits in 5xFAD can be the Aβ aggregation that closely relates to the impairment of synaptic plasticity and degeneration of neurons. This cascade of events yields abnormal neuronal activity that in turn disturbs homeostatic firing in the hippocampus of the AD brain.^42^ Therefore, it is imperative to examine the formation of synapses in 5xFAD mice with various treatments. The ultrastructure of synapses was examined by electron microscopy of brain samples of 5xFAD mice with various treatments. Importantly, the treatment of 5xFAD mice with Fe65-EXO-Cory-B significantly augmented the formation of synapses compared with vehicle- or Cory-B- treated 5xFAD mice (Fig. 5k, l), suggesting that Fe65-EXO-Cory-B could effectively enhance the formation of synaptic plasticity between neurons in the hippocampus of the AD brain, rescuing the learning and memory impairment in 5xFAD mice.

## Discussion

AD has created a catastrophic public health issue with the aging global population. Immediate intervention to control the current epidemic scenario is vital. The key hallmarks of AD include aggregation of β-amyloid peptides and hyperphosphorylated tau in NFTs, with typical and distinctive clinical symptoms such as progressive deterioration of episodic memory and executive functions. In the context of therapeutic intervention, the available drugs approved by the Food and Drug Administration (FDA) for AD patients, namely NMDA receptor 2 modulator (e.g., memantine), and cholinesterase inhibitors (e.g., rivastigmine, donepezil, and galantamine) can help to improve only quality of life and increase life expectancy. They cannot halt AD progression. There is a demand for novel therapeutic approaches to AD treatment. When developing a novel therapeutic intervention for AD treatment, it is crucial to consider the selective nature of the barrier around the brain, the BBB, that prevents entry of hydrophobic therapeutic drugs to the brain. Interestingly, our target drug, Cory-B, a natural autophagy inducer, has been found to effective in AD therapy. Nonetheless its delivery to the brain is challenging, as evidenced by its lesser bioavailability in the brain of an AD mouse model^11^ that required an appropriate carrier to facilitate its brain delivery for potential AD treatment.

Recently, exosomes have gained massive attention as a drug delivery carrier, owing to their ability to cross the BBB, participate in the intracellular communication by carrying various cargoes including nucleic acids, and proteins. Importantly, exosomes can be found in the blood and can be utilized as a theranostic platform.^12,19^ Also, their surface is tunable and can be engineered to express the ligands for targeting receptors overexpressed in disease conditions. In the present study, APP was utilized as a target receptor, generally found to be overexpressed in neuron cells in the brain of AD patients (Supplementary Fig. S18a–c). A neuronal adapter Fe65 binds to APP, therefore, engineered exosomes were generated by OE of Fe65 in hippocampus neuronal cells via transfection of pCI-Fe65 plasmid, followed by isolation and characterization of exosomes expressing Fe65 on their surface (Fig. 1a–p, Supplementary Fig. S1a–c). Intriguingly, Fe65 has also been implicated in different functions such as insulin based potentiation of neurite outgrowth,^43,44^ which could be further analyzed in the context of AD therapeutics. It is worth noting that Fe65-EXO could efficiently bind or be internalized by neuronal cells with APP overexpressed at a high magnitude, suggesting a capacity of Fe65-EXO to target APP overexpressed neuronal cells (Fig. 2a–p). Interestingly, autologous cells showed no significant change in viability upon treatment with Fe65-EXO, whereas there was a slight decline in that of heterologous neuronal cells upon treatment with Fe65-EXO, suggesting suitability of autologous exosomes for drug delivery in general or targeted drug delivery (Fig. 2q, r).

For decades, dysfunction of the autophagy-lysosomal degradation pathway has been put in association with AD progression.^28,29^ Interestingly, a relationship between exosomes and autophagy that is considered a key protein degradation pathway functioning to clear misfolded proteins in various neurodegenerative diseases including AD, has been studied. Additionally, several pro- and anti-inflammatory molecules have been found in exosomal cargo, which may play a role in regulating the central nervous system (CNS) inflammatory problem.^45^ It is logical to investigate the ability of Fe65-EXO-Cory-B to induce autophagy in a neuronal cell-based AD model. As expected, the Fe65-EXO-Cory-B could induce autophagy as shown by the augmented level of autophagy markers, SQSTM1 and LC3B-II in neuronal cells. Also, the delivery of Cory-B via Fe65-EXO to neuronal cells induced the augmented autophagy compared with Cory-B alone; mediated via BECN1, ATG5 and ATG7 (Fig. 3a–d, Supplementary Figs. S2a, b, 3a–h, and 4a–d).

The BBB has been proved leaky in patients with AD and related to decline in cognition, which indicates that a compromised BBB may be a crucial component of AD pathogenesis.^46,47^ Several groups have reported the capability of exosomes to cross the BBB in in vivo models of various diseases such as glioma and AD.^48^ Therefore, it was intriguing to know which route of administration of Fe65-EXO-Cory-B would show the highest bioavailability in the BBB. Our results demonstrated that Fe65-EXO-Cory-B injected intravenously could reach the target site, evidenced by APP overexpressed neuronal cells in the AD mice brain (Fig. 4a–t), without any noticeable toxicity to major organs including the brain in an AD mice model (Supplementary Figs. S14, 15, 16). Nevertheless other routes of delivery can be evaluated to further improve bioavailability such as the intranasal route since it offers a practical and non-invasive approach to bypass the BBB for therapeutic delivery to the brain and spinal cord.^49^

Until now, we understood that Fe65-EXO-Cory-B could be efficiently delivered to neuronal cells with overexpressed APP in the AD mice brain. Nonetheless, to further establish that it can ameliorate AD pathogenesis, we needed to examine the effect of Fe65-EXO-Cory-B on cognitive and locomotor behavior in an AD mice model. It is evident from various behavior experiments such as the rotarod test, open field test, CFC test, and MWM test that Fe65-EXO-Cory-B improves cognitive function in AD mice (Fig. 5a–l, Supplementary Fig. S17). Collectively, it is appropriate for the engineered exosome-based targeted drug delivery demonstrated in this study to be applied for other brain diseases such as glioma, Parkinson’s disease, and amyotrophic lateral sclerosis. Although hippocampus neuronal cells have been widely utilized as a source of exosome and their genetic engineering, several other cell types including macrophages or stem cells like mesenchymal stem cells or induced pluripotent stem cell (iPSC)-derived neuronal cells could also be explored as a potential source of efficient exosome for targeted delivery. As a payload, several other drugs as well as their combination can be potentially loaded into exosomes for brain delivery.

Although our findings reveal the potential for engineering neuronal cell-derived exosomes to be an innovative system for brain drug delivery, further studies are still required. Potential studies including the establishment of the delivery route of exosomes via the olfactory region to the brain are necessary for efficient application. Additionally, it is worthy to examine the feasibility of an engineered exosome-based delivery system for clinical translation as well as the possible side effects.

## Materials and methods

### Chemical and reagents

Corynoxine-B (Aktin Chemicals Inc, Cat# APC-459), 3-methyladenine (3-MA) (Cat# M9281) and chloroquine (CQ) (Cat# C6628) were purchased from Sigma–Aldrich. Torin1 (Cat# 2273) was purchased from BioVision. Bafilomycin A1 (Baf A1) (Cat# S1413) was purchased from Selleckchem. HRP-conjugated goat anti-mouse IgG (Cat# 115-035-003) and goat anti-rabbit IgG (Cat# 111-035-144) secondary antibodies were purchased from Jackson ImmunoResearch Laboratories. Atg7 (mouse) siRNA (Cat# L-049953-00-0005), Becn1 (mouse) siRNA (Cat# L-055895-00-0005), and non-target siRNA were purchased from Dharmacon. Alexa Fluor® 594 goat anti-rabbit IgG (Cat# A-11012) Alexa Fluor 488 Goat Anti-Mouse IgG (Cat# A11001), Alexa Fluor 488 Goat anti-Rabbit IgG (Cat# A11008) were purchased from Life Technologies. Dulbecco’s Modified Eagle Medium (DMEM) (Cat# 11965084, Gibco), fetal bovine serum (FBS) (Cat# 10500064, Gibco), Lipofectamine 3000 reagent (Cat# L3000015, Invitrogen), 2-Mercaptoethanol (Cat# M6250), Opti-MEM medium (Cat# 31985070, Gibco), Lipofectamine RNAiMAX reagent (Cat# 13778030, Invitrogen), RIPA lysis buffer (Cat# 9803, Cell Signaling Technology), protease inhibitors (Cat# HY-K0011, MedChemExpress), phosphatase inhibitors (Cat# A32957, Pierce), BCA assay reagent (Cat# 23225, Pierce), Triton X-100 (Cat# T9284, Sigma), Cryomatrix (Cat# 6769006, Thermo Scientific), TurboFect (Cat# R0531), Medical X-ray film (Cat# CK04, FUMINGWEI), PSN-Antibiotic Mixture (Cat# 15640055), 10X Tris/Glycine/ SDS buffer (Cat# 161-0772), PVDF membrane (Cat# 10600023), 10X Tris/Glycine buffer (Cat# 161-0771), Protein ladder (Cat# 26616), 4 x Laemmli buffer (Cat# 161-0747), 2X Laemmli sample buffer (Cat# 161-0737), Fluorescence mounting media (Cat# S3023), paraformaldehyde (PFA) (Cat# P6148), ECL kit (Cat# 34580, SuperSignal, Thermo Scientific), glutaraldehyde (Cat# G5882, Sigma), sodium cacodylate buffer (Cat# 11654, Electron Microscopy Sciences), NaCl (Cat# J21618-A7, USB), NP40 (Cat# 74385, Sigma), glycerol (Cat# 15514-011, Invitrogen), Tris (Cat# 75825, USB), EDTA (Cat# E9884, Sigma), Thioflavine S (Cat# T1892, Sigma), LysoTracker® Red DND-99 (Cat# L-7528) and FluorSave reagent (Cat# 345789, Calbiochem). Total Exosome Isolation (TEI) reagent (ThermoFisher Scientific, Cat# 4478359). The details of antibodies used in this study are listed in Supplementary Table S1.

### Animals

Animal related experiments were conducted as per protocols approved by the Committee on the Use of Human and Animal Subjects in Teaching and Research (HASC) at the Hong Kong Baptist University (HKBU) (# HASC/20-21). The research personnel who performed the experiments for the research study gained approvals as per the health department policy, Hong Kong government and the animal license was approved before starting the experiments (20–28), (22–33), (22–34) in DH/HT&A/8/2/6 Pt.5. All transgenic mice colonies were housed and maintained at the Laboratory Animal Unit of the Jockey Club School of Chinese Medicine, HKBU. The 5xFAD mice and their littermates wild-type (WT) C57BL/6 N were purchased from Jackson Laboratory that over-express mutant human APP (695) with the London (V717I), Florida (I716V) and Swedish (K670N, M671L), familial AD (FAD) mutations M146L and L286V along with human PS1 harboring under mouse Thy1 promoter. The 3XTg-AD mice, with a background of C57BL6/129SVJ, with 3 mutant overexpression’s (K670M/N671L, M146V, P301L) were purchased from Jackson Laboratory.

### Cell culture

Mouse neuroblast Neuro-2a cells (N2a), and N2a cells expressing Swedish-mutant APP (N2aSwedAPP) and mouse hippocampal neuronal (HT22) cells were used. All the cells were grown in standard culture medium containing DMEM, supplemented with 10% FBS and 1% penicillin-streptomycin solution.

### Treatment of drugs

5xFAD and C57BL/6 N mice were randomly allocated to WT-Vehicle, Tg-Vehicle, Tg-Cory-B (20 mg/kg), Tg-Fe65-EXO, Tg-Fe65-EXO-Cory-B (20 mg/kg), or Tg-Fe65-EXO-Curcumin (20 mg/kg) for the treatment schedule and behavioral experiments. All drug formulations were administered intraperitoneally (IP) or intravenously (IV) to the 5xFAD and C57BL/6 N (Wild-Type; WT) mouse models on alternate days for 45 days. Treatment with all drugs and exosome formulations was for 24 h. N2a and HT22 cells were used for most experiments in cell culture. The drugs Cory-B (20 µM), Torin1(250 nM), and Curcumin (10 µM) were used at the mentioned concentration in all cell culture experiments. 10–20 µM of Chloroquine (CQ) was utilized for 24 h for inhibiting lysosomal fusion and 5 mM of 3-Mehtyl adenine (3-MA) used for the PI3K inhibition assay.

### Genetic modification of hippocampus neuron cells to produce Fe65-engineered exosomes

Fe65-engineered exosomes were produced by transfecting HT22 cells with pCI-Fe65 plasmid.^50^ Briefly, cultured HT22 cells were transiently transfected with the pCI-Fe65 plasmid using Turbofect reagent for at least 48 h to obtain a 70–80% transfection efficiency. Next, the media was collected for Fe65-engineered exosome isolation (described in next section) and cells scraped from the plate to collect cell lysate for determination of the percentage of Fe65 OE using immunoblot.

### Isolation of exosomes

Exosomes were isolated from the cell culture conditioned medium using Total Exosome Isolation (TEI) reagent as described previously.^12^ Briefly, extracellular medium was collected from HT22 or Fe65 OE-HT22 cells cultured for 24 h, and the centrifuged at 2000 × *g* for 30 min. Next, the supernatant was mixed with half volume of TEI reagent and incubated at 4 °C overnight, followed by centrifugation at 10,000 × *g* for 1 h at 4 °C. In its working mechanism, TEI reagent binds to water molecules and forces less-soluble components such as vesicles out of solution, allowing them to be collected by a swift, low-speed centrifugation. The supernatant was discarded, and the pellet (which contains exosomes) was diluted with 1 × phosphate buffer saline (PBS) for NTA, with 1X radioimmunoprecipitation assay buffer and cocktail inhibitor for WB or fixed with paraformaldehyde (PFA) for transmission electron microscope (TEM) and immunogold-EM. The zeta potential of exosomes was analyzed by using Malvern Zetasizer Nano ZS.

### Determination of size and concentration of exosomes by NTA

Size distribution and concentration of exosomes was examined using a Malvern NTA machine. Briefly, a laser beam at the wavelength of 405 nm was utilized on the exosome solutions (volume = 500 ml) inserted into the sample chamber. Videos of exosomes in motion were captured and measured by NTA software (version 2.2, NanoSight NS300).

### Evaluation of exosomes by TEM

The size, shape, and morphology of exosomes were identified by TEM analysis. Briefly, a negative staining technique was utilized to view the exosomes. A drop (30 μl) of the suspension of exosome in filtered PBS was poured on carbon-coated electron microscopy (CCEM) grids, placed on parafilm, and incubated at room temperature for 10 min. Then, it was transferred to a drop of Uranyless® solution for 1 min and left to be air dried, and eventually the surplus stain was blotted away. Imaging of the exosomes processed on the CCEM grid was performed by using a TEM machine (FEI Spirit 120 kV LaB6 Electron Microscope) at 100 kV [at the University of Chicago Advanced Electron Microscopy Core Facility (RRID: SCR_019198)].

### Immunogold electron microscopy of exosomes

The presence of CD63 and Fe65 proteins on exosomes was validated using immunogold-EM. Briefly, the exosome suspensions (fixed with 2% PFA) were adsorbed CCEM grids, placed on parafilm for 5 min. Next, the grids were washed for 15 min in solution of 0.05 M filtered glycine in PBS, followed by incubation in wash buffer (0.1% BSA in PBS), and blocking of exosome samples with 1% donkey serum for 30 min. The grids were incubated for 24 h with a mouse anti-CD63 or anti-Fe65 primary antibody (dilution = 1:100). Following six washes and incubation with 10 nm gold-conjugated donkey anti-rabbit antibody for 2.5 h, the grids were washed, post-fixed in 2.5% glutaraldehyde in PBS, and washed with PBS and distilled water. Excess fluid was blotted with filter paper, and the samples dried for 30 min before imaging using a TEM machine [at the University of Chicago Advanced Electron Microscopy Core Facility (RRID: SCR_019198)].

### Loading of Corynexin-B in exosomes and their evaluation

Loading of Cory-B in Fe65-EXO was carried out using the sonication method and loading efficiency calculated using UV-Visible spectroscopy and liquid chromatography-mass spectroscopy (LC–MS). Briefly, for drug loading into exosomes by sonication, an equal amount of Cory-B and exosome was mixed and sonicated (20% amplitude and 6 cycles of 30 s on/150 s off). After sonication, the Cory-B and exosome solution was incubated at 37 °C for 60 min. Excess free drug was separated from EXO-Cory-B by size exclusion chromatography using a Sephadex G25 column. For further validation, the samples were prepared in high performance liquid chromatography (HPLC) grade methanol, and an internal control used for analysis using Agilent 1290 (Agilent Technologies) UHPLC system. Briefly, 2 ml of sample was injected into C18 column (1.7 µm 2.1 × 100 mm) (Acquity UPLC BEH) for its separation at 40 °C. The mobile phase constituted 0.1% formic acid (A) in water and 0.1% formic acid in ACN (B), with a constant flow rate of 0.1 ml/min. The MS and MS/MS data were obtained by an Agilent 6540 Q-TOF mass spectrometer (Agilent Technologies) equipped with a jet stream ESI source in positive ion mode. The data was analyzed by Mass Hunter (Agilent Technologies) B.03 software and for all mass peaks, the mass spectral data were analyzed at a range of 100e1700 m/z.

### Western blotting

Concentration of protein in cell lysates, or exosomes was measured by Bradford Assay (Bio-Rad, USA). Equal amount of protein samples from cells or exosomes were loaded and separated on the SDS-gel electrophoresis, followed by their transfer to the PVDF membrane at room temperature. Next, the membrane containing protein samples were blocked with 5% non-fat milk in TBS-Tween 20 (0.1%) (TBST) for 1 h at room temperature and incubated with primary antibodies overnight at 4 °C. After washing with TBST for 3 times, membranes were incubated for 1 h at room temperature with HRP-conjugated secondary antibodies. The desired bands were visualized by enhanced chemiluminescence (ECL) reagents.

### Dot-blot immunoassay

Ten microliter of protein sample (1 μg/μL) was added to a nitrocellulose membrane, fitted in the Bio-Rad Bio-Dot system. Next, the membrane was blocked with 5% non-fat milk/TBST for 1 h, followed by incubation with primary antibodies for 1 h. Eventually, the membrane was incubated with HRP-conjugated secondary antibodies for 30 min. The signal of dot-blot was developed by Western bright Quantum HRP substrate and determined by the Bio-Rad ChemiDoc™ Imaging Systems.

### Labeling of exosomes

Exosomes were labeled with Exo-Green Exosome Protein Fluorescent Labeling reagent (Cat# EXOG200A-1, System Biosciences). Briefly, 50 µL 10x Exo-Green was added to 500 µL volume of exosome solution in 1XPBS (200 µg protein) and flicked for mixing. Next, the exosome solution was allowed to be incubated for 10 min at 37 °C, then FBS added to stop the labeling reaction. The labeled exosome solution was placed at 4 °C for 30 m, followed by centrifugation for 3 min. at 14,000 rpm. The supernatant containing excess label was removed, and the labeled exosome pallet resuspended in PBS for subsequent monitoring.

### Analysis of uptake of Fe65-EXO by autologous and heterologous APP OE neuron cells

Approximately 30,000 cells per well of recipient cells (HT22, APP OE-HT22, N2a, and APP OE-N2a) were cultured on the chamber slide (Lab-Tek, Thermo Scientific, USA) for 24 h. The following day, cells were washed twice with PBS and replenished again with medium supplemented with 250 μg Fe65-EXO (100 μl) and maintained for 24 h. Afterward, the recipient cells were washed with PBS (three times) and fixed with 4% PFA on ice for 30 min, then washed again with PBS. The APP OE was tagged with red color, and internalized Exo-Green labeled exosomes showed green. Then, stained recipient cells on the slide were covered with a thin layer of Vectashield medium containing DAPI and viewed under a confocal microscope.

### Cell viability assay

The viability of cell was measured via MTT assay. Briefly, HT22 or N2a neuronal cells (10,000 cells per well) were seeded in a 96-well microtiter tissue culture plate. After 24 h of culture, the medium was removed, and neuronal cells were washed with PBS. To determine the optimum dose of exosomes, the effect of different amount of exosomes (0, 0.01, 0.1, 1, 5, 10, 20, 50, 100, 200, 400, and 600 μg) on neuronal cells were examined. Subsequently, neuronal cells were incubated with 20 μL of Control EXO, or Fe65-EXO for 24 h; control was treated with vehicles (PBS in medium). After incubation, medium in neuronal cells’ culture was removed, and MTT (5 mg/mL in DMSO) solution was added to each well. Cells were then incubated at 37 °C for 4 h. Next, medium in each well was removed, and 150 µL of DMSO was added to each well and mixed thoroughly. Finally, the plates were incubated at 37 °C for 10 min, and absorbance was measured on a microtiter plate reader at 570 nm.

### Immunocytochemistry

ICC staining was performed in the above-mentioned cell lines. Cells seeded on glass cover slides in a 24-well-plate (Falcon, U.S.A.) and designated treatments applied after 24 h. After treatment, cells were fixed with 3.7% PFA (Sigma, U.S.A.) for 15 min at room temperature. Neuronal cells were allowed to be incubated with respective target primary antibodies for 18 h. The secondary antibody conjugated with FITC was added to cells for 1 h. Cover slides were picked from the wells and cell-side-down covered on a glass slide, mounted with FluorSave reagent (Calbiochem, U.S.A.). Fluorescent images were recorded using a confocal microscope (Olympus).

### Immunohistochemistry

PFA-fixed, frozen, and cryomatrix-embedded brains of 5xFAD and WT mice were prepared and sectioned, around 5–30 μm, mounted on coated slides for IHC. For each region, three sets of sections were processed for immunostaining analysis. Primary antibodies for APP and other proteins (Supplementary Table S1), along with EXO-Green labeled exosomes were examined. Sections through each anatomic region of interest were captured, and a threshold optical density was obtained to discriminate staining from the background. Image analysis was carried out by Image J analysis (NIH) software.

### Evaluation of cellular ultrastructure by EM

HT22 or N2a cells were seeded in 100 mm dish and cultured in DMEM media with 10% FBS and 50 units of PSN mixture as supplements. After reaching a confluence between 60 and 70%, HT22 or N2a cells were treated with drugs including Cory-B (20 µM), Torin1 (250 nM), Fe65-EXO, Fe65-EXO-Cory-B, or Fe65-EXO-Curcumin (10 µM) for a period of 24 h. After treatment, the medium was discarded, and cells were fixed with 2.5% glutaraldehyde in 0.1 M sodium cacodylate buffer for 1 h at room temperature. The fixed cells were collected via scraping from the cell culture plate into a centrifuge tube and then centrifuged. The pellet of the fixed cells was dissolved and rinsed in 0.1 M sucrose in cacodylate buffer and post-fixed with 1% osmium tetroxide for dehydration. The dehydrated cells were embedded in Poly/Bed® 812 for ultra-thin slicing to examine their components, followed by sectioning using an ultramicrotome with thickness around 100 to 200 nm before being fixed in carbon-coated electron microscopy grids and viewed under a Philips CM100 transmission electron microscope at the QMH Electron Microscope Unit, Queen Mary Hospital, University of Hong Kong, Hong Kong.

For the examination of synapse formation in the mice brain, the tissue samples were fixed, dehydrated, embedded, sectioned, stained with uranyl acetate, followed by mounting on the grid for electron microscopy examination.

### Autophagy assay

HT22 or N2a cells were cultured on the cover glass in a 24-well plate. After 60 to 70% confluence was reached, HT22 or N2a cells were transiently transfected with GFP-LC3 plasmid or Tandem fluorescent tagged-LC3 plasmid using Turbofect reagent (R0531) to obtain a 70–80% transfection efficiency for 24 to 48 h. Next, cells were treated with drugs Cory-B (20 µM), Torin1(250 nM), Fe65-EXO, Fe65-EXO-Cory-B, or Fe65-EXO-Curcumin (10 µM) and negative control CQ (20 µM) for 24 h. Next, the medium was discarded, and the cells were washed twice with 1XPBS. Cells were then fixed with 4% PFA for 15 min and those in the cover glass embedded in the slides with the DAPI rich mounting media. Slides were viewed under the confocal microscope (Leica TCS SP8, Weitzlar, Germany) and all acquired images analyzed using Image J software.

### In vivo exosomal tracking

5xFAD mice were randomly allocated to drug treatment, Tg-Vehicle-Exo, Tg-Fe65-EXO, Tg-Cory-B (20 mg/kg), Tg-Fe65-EXO-Cory-B (i.v.) (20 mg/kg) or Tg-Fe65-EXO-Cory-B (i.p.) (20 mg/kg) for live animal imaging. After single dose treatment of labeled exosomes, animals were anesthetized using 2 or 3% chloral hydrate as per body weight. Fluorescence images at different time points (20, 35, 80, 150, 180, 240, 270 and 360 min.) were gained with the IVIS Lumina XR under a laser power of 70–80 V. A filter (excitation at 488 nm, and emission at 550–650 nm) was applied to acquire fluorescent images. After all imaging was completed, blood samples were taken, and mice then sacrificed, and all organs removed for further analysis. Organs were imaged using IVIS Lumina XR to collect fluorescence images.

### Analysis of behavior in AD mice

#### Rotarod test

Motor coordination and balance were examined in AD mice using rotarod apparatus. Each mouse was allowed to be acclimatized (five trials, 10 min apart) followed by two test trials after an hour by placing the mouse on the rotarod (speed = from 4 to 40 rpm over 300 s). After two successive trials for each mouse, the mean latency time to fall from rotarod was recorded (Harvard apparatus, SeDaCom v2.0.000).

#### Open field test

An open field-testing paradigm was applied to examine the locomotor and exploratory behavior of AD mice. Briefly, mice were first placed in the center of an open field, and exploratory behavior of them were observed for a period of 5 min. Cages were regularly cleaned with ethanol after every session. ANYMAZE (Stoelting) software was used to measure the distance traveled, center rears and wall rears were used as measures of locomotion, while time spent in the central square, stretch attend postures, freezing, grooming, and defecation and urine spots, were used to measure exploratory and anxiety-like behavior.

#### Contextual fear conditioning test

Fear conditioning tests were performed in a 30 × 24 × 21 cm chamber that was placed in a 110× 50 × 60 cm plastic cabinet. In the training session, mice were placed in a chamber and given a two-minute period to explore the chamber. When 2 min ended, an audio tone (conditional stimulus: 5 kHz,70 dB) was emitted for 28 s followed by a foot shock (unconditional stimulus: 0.5 mA) from the metal grid on the floor for 2 s. Foot shock intensity, which was the minimal applicable intensity required to elicit a response, was determined ahead in a preliminary test carried out on a separate cohort of mice. On the next day, both contextual and cued conditioning tests were performed. In the contextual fear session, mice were placed back to the conditioning chamber and remained for 5 min without any shock or tone. The time of freezing was recorded as an index of contextual memory. After 3 h of rest, the tone-associated, cued conditioning tests were performed using ANYMAZE (Stoelting) animal tracking software. Time freezing during and between the audio tones was recorded as an index of cued memory.

#### Morris water maize test

All mice were first tested in a straight swim pretraining protocol. Then a hidden platform that was placed in the center of one quadrant of the pool was applied for all mice to go through reference memory training for a period 6 days, with 4 trials per day. After the last trial of 6th day, the platform was removed from the pool. After another 24 h, each mouse was tested for one 60-s swim “probe trial” on the 7th day. Escape latency (in seconds), length of swim path and swim speed, were recorded using an on-line ANYMAZE (Stoelting) real time video tracking system. Behavioral data were analyzed using ANYMAZE (Stoelting) software.

#### Novel object recognition test

The 5xFAD mice model was evaluated for memory function and recognition memory using a Novel Object Recognition Test. Prior to the test, the animals were given one day to familiarize with the test environment by freely exploring the box. On Day 1, the mice were trained on two known objects placed at an equal distance from the center. On Day 2, one of the objects was replaced with a novel object of different shape. The animal’s exploring behavior on the novel object was then recorded for 5–10 min using an on-line ANYMAZE real time video tracking system (Stoelting). The recognition index percentage was calculated with ANYMAZE software (Stoelting Co., Wood Dale, IL, USA).

### Pharmacokinetic analysis

The time-dependent concentration of Cory-B was measured in the brain and plasma samples of study animals treated with Cory-B, and Fe65-EXO-Cory-B. 5XFAD mice were used for the study according to the regulation of the Animal Ethics Committee. Mice were randomly separated and grouped for the time point and *N* = 3 in each group were taken for pharmacokinetics study of the target drugs. Animals were allowed to fast overnight before the experiment with free water access. On the day of experiment, Cory-B and Fe65-EXO-Cory-B were administered intraperitoneally. Brain extraction and blood collection were carried out into a tube (heparinized) at 5, 10, 20, 40, 80, 120, 240, and 300 min, after administration, followed by centrifugation at 4000 × *g* for 10 min at room temperature. Eventually, the supernatant was transferred to a fresh tube and stored at −20 °C, until it is used for analysis.

### Analysis of gross necropsy and histopathology

5XFAD and 3xTg-AD mice with various treatments were examined for the external body surface, all orifices and the thoracic, abdominal, and cranial cavities and their contents. Various body organs including heart, liver, kidneys, spleen, brain, and lungs were examined after dissection, followed by preservation of respective tissue samples (in 10% formalin) from each mouse in the single dose toxicity study for histopathological examination. Gross lesions were also examined and recorded in all groups. The preserved tissues were molded in Cryomatrix^TM^ followed by the cutting and staining of 10 μm thick sections with hematoxylin and eosin (H&E). Histopathological examination was conducted under Nikon E600 light microscope. The investigator responsible for the histopathological observations was blinded to the treatment assignment of each animal.

### Analysis of dendritic spines via Golgi staining

After perfusing the animals with PBS, the brains were bisected sagittally, and one half was fixed in Solution AB for two weeks at room temperature. The solution was changed twice. Solution AB was replaced with Solution C for three days. The tissue was washed twice with distilled water and sectioned on a cryotome. Brain slices were placed on coated slides to remove Solution C and air-dried for two days. The slides were washed twice with water and incubated in Solution DE for 10 min. The slides were washed twice with water and incubated in a series of ascending concentrations of alcohol to dehydrate the tissue. The slides were cleared with xylene and mounted with coverslips using mounting media. The spines and dendrites were photographed under a microscope and analyzed for spine density and morphology using NIH Image J software.

### Statistical analyses

Data were presented as the mean ± S.E.M. Histograms were produced for the evaluation of the data normality. The nonparametric Kruskal–Wallis H test was conducted, followed by the post-hoc analysis via Mann–Whitney U test under the situation of abnormally distributed data. Further, for statistical analysis, parametric one-way analysis of variance (ANOVA) was performed, followed by post-hoc comparison of the means using Bonferroni’s or Dunnett’s methods, if the normally distributed data. Behavior data was analyzed by two-way ANOVA for repeated measures with “treatment” and “day” and their interactions as fixed. The respective *P* values for significance level have been mentioned in the figures’ legends. All analyses were performed using SigmaStat software or GraphPad Prism version 6.

### Supplementary information

Supplementary Data File

## Data Availability

All data required for the assessment and conclusions in this research have been included in this manuscript and/or the supplementary data file. Further data or queries can be available from the corresponding authors upon reasonable request.
