# Supplementary material for: Fe65-engineered neuronal exosomes encapsulating corynoxine-B ameliorate cognition and pathology of Alzheimer’s disease
Source: Signal Transduct Target Ther. 2023 Oct 23;8:404. doi: 10.1038/s41392-023-01657-4 (PMC10590775; doi:10.1038/s41392-023-01657-4)
Supplement: Supplementary file 1 — Supplementary Data File [file 41392_2023_1657_MOESM1_ESM.docx]

Supplementary Materials for

Fe65 Engineered Neuronal Exosomes Encapsulating Corynoxine-B Ameliorate Cognition and Pathology of Alzheimer’s Disease

Ashok Iyaswamy^#,*^, Abhimanyu Thakur^#,*^, Xin-Jie Guan, Senthilkumar Krishnamoorthi, Tsz Yan Fung, Kejia Lu, Isha Gaurav, Zhijun Yang, Cheng-Fu Su, Kwok-Fai Lau, Kui Zhang, Roy Chun-Laam NG, Qizhou Lian, King-Ho Cheung, Keqiang Ye, Huanhuan Joyce Chen^*^, Min Li^*^

^#^Authors contributed equally.

^*^Correspondence to:

Prof. Min Li, E-mail: [limin@hkbu.edu.hk](mailto:limin@hkbu.edu.hk), Tel: + 852-3411 2919, Fax: +852-3411 2461

Dr. Huanhuan Joyce Chen, E-mail: [joycechen@uchicago.edu](mailto:joycechen@uchicago.edu), Tel: +1-773-834-4465.

Dr. Ashok Iyaswamy, E-mail: [iashok@hkbu.edu.hk](mailto:iashok@hkbu.edu.hk), Tel: +852-3411-2061

Dr. Abhimanyu Thakur, E-mail: [abhimanyu@uchicago.edu](mailto:abhimanyu@uchicago.edu), Tel: +1-773-219-4550

**This PDF file includes:**

Figures. S1 to S18

Tables S1


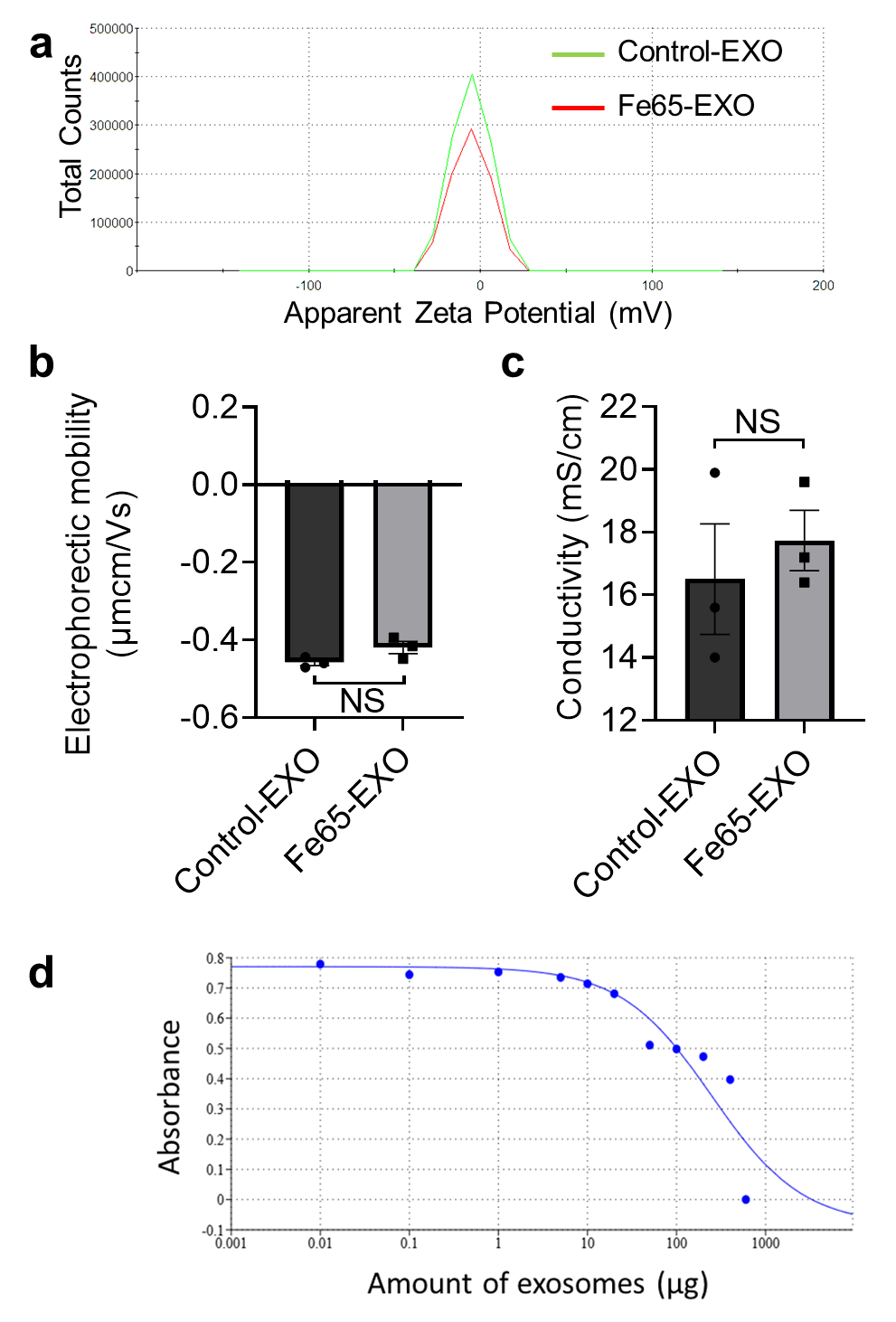


**Figure. S1. Effect of Fe65 engineering on the exosome membrane property.** Representative **(a)** apparent zeta potential distribution, **(b)** bar graph showing electrophoretic mobility, and **(c)** bar graph showing conductivity of exosomes before and after engineering with Fe65. **(d)** Representative graph showing the exosomes dose (0, 0.01, 0.1, 1, 5, 10, 20, 50, 100, 200, 400, and 600 μg) dependent effect on the cell viability of HT22 neuronal cells. The IC50 value was found to be 248.76 μg.


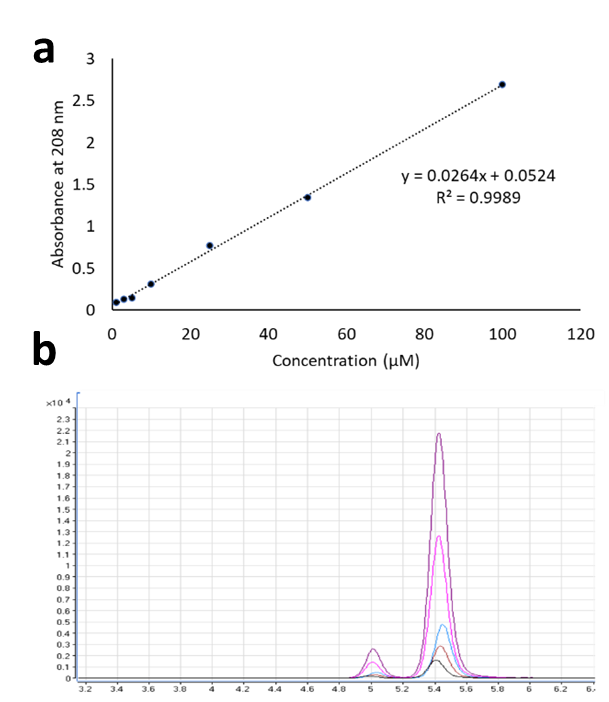


**Figure. S2. Loading of Cory-B in Fe65-EXO via sonication method. (a)** Representative standard curve based on the UV-Visible spectra of various concentrations of Cory-B vs. absorbances at 280 nm wavelength. **(b)** Representative LC-MS curves of various concentrations of Cory-B.


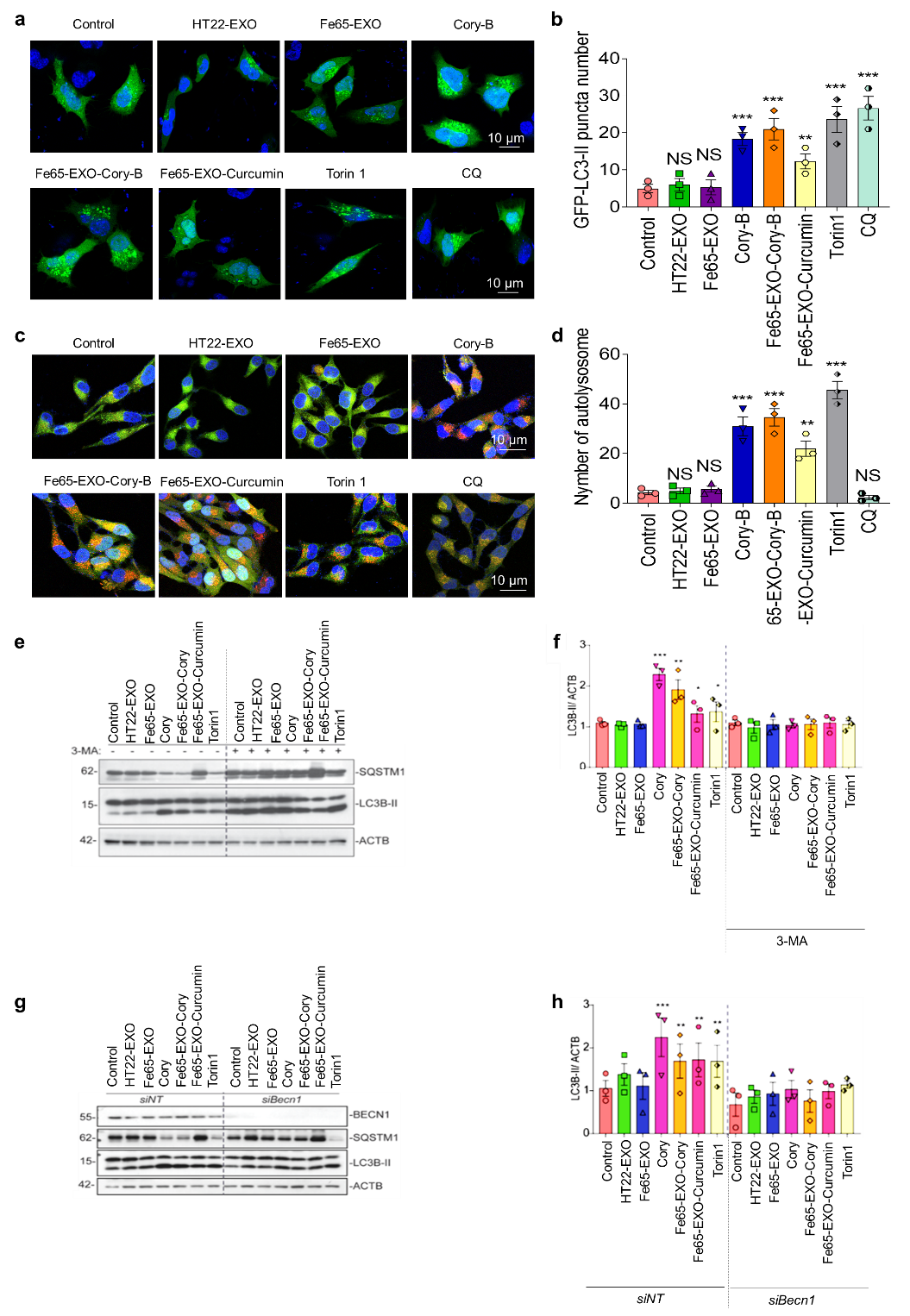


**Figure. S3. Delivery of Cory-B by Fe65-EXO caused** **BECN1-dependent augmented autophagy in neuronal cells.** **(a, b)** Representative immunofluorescence staining showed the pattern of tf-LC3 (RFP puncta) in N2a cells, and the corresponding bar graphs, respectively. N2a cells were transfected with tf-LC3 plasmids for 24 hr before various treatments. Scale bar: 10μm. **(c, d)** Representative immunostaining images show the pattern of GFP-LC3 in N2a cells. N2a cells were transfected with GFP-LC3 plasmids for 24 h, followed by various treatments, and the corresponding bar graphs, respectively. Scale bar: 10 μm. **(e-h)** Representative immunoblots showing the protein level of SQSTM1 and LC3B-II and corresponding bar graphs showing their quantification in N2A cells with (g, h) treatment of 3-MA or (i, j) knockdown of BECN1. In b, d, f, and h; data were shown as mean ± SEM (N=3). Significance level: ^***^P<0.001, ^**^P<0.01, ^*^P<0.1; treatment groups vs. control group.


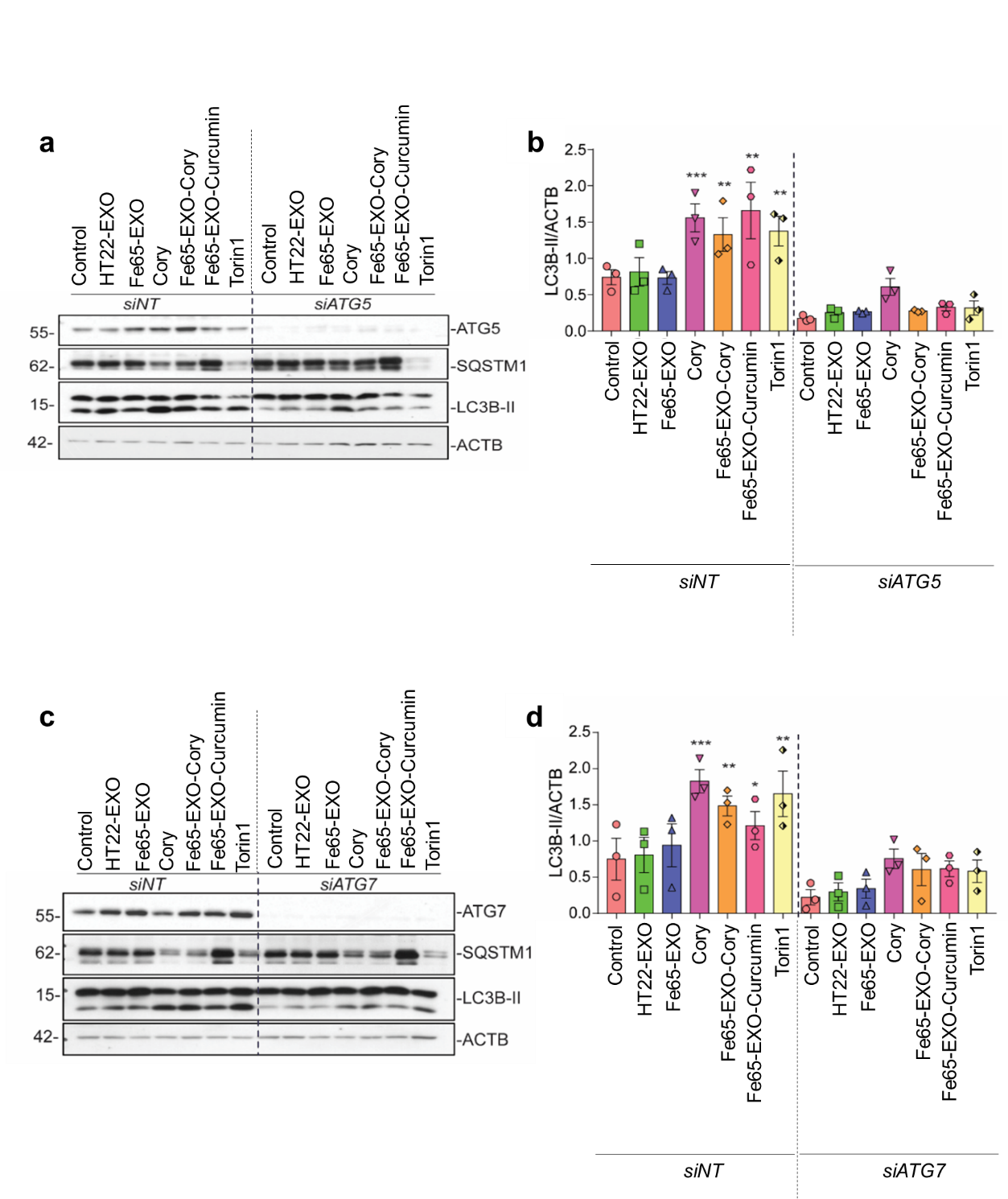


**Figure. S4. Cory-B loaded Fe65-EXO enhances the autophagy in neuronal cells** **independent of ATG5 and ATG7.** Representative western blots showing the protein levels of SQSTM1 and LC3B-II and bar graphs showing their quantification in N2A cells with **(a, b)** knockdown of ATG5 or **(c, d)** knockdown of ATG7. Data are shown as mean ± S.E.M. Control (vehicle) vs. Control-EXO, Control vs Fe65-EXO, Control vs Cory-B, Control vs Fe65-EXO-Cory-B, Control vs Fe65-EXO-Curcumin, Control vs Torin1, Significance level: ^***^P<0.001, ^**^P<0.01, ^*^P<0.1.


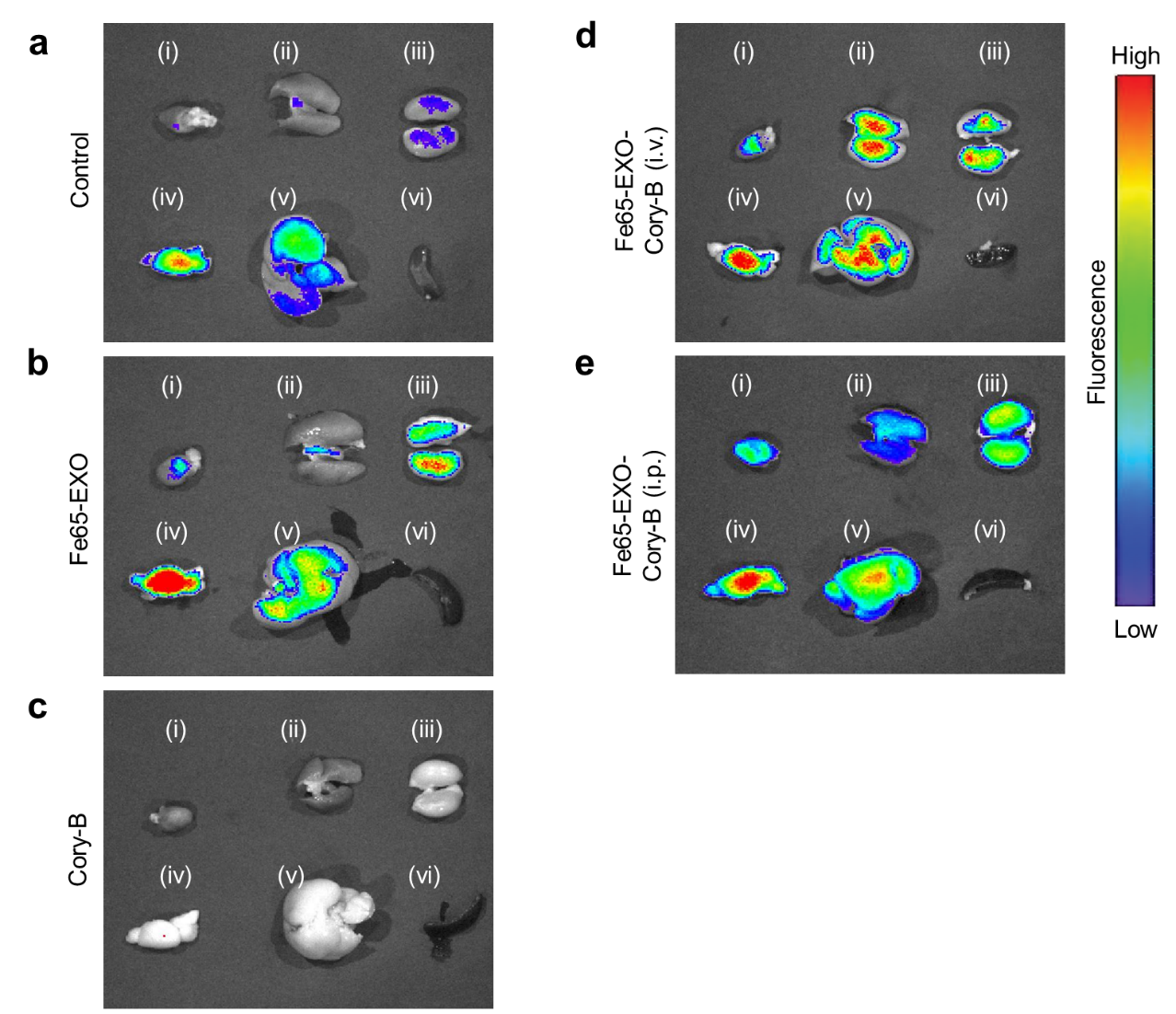


**Figure. S5. Biodistribution of Fe65-EXO-Cory-B in different organs of AD mouse.** *Ex vivo* fluorescence images showing the distribution of **(a)** control exosomes, **(b)** Fe65-EXO, **(c)** Cory-B, and **(d, e)** Fe65-EXO-Cory-B in the (i) heart (ii) lung (iii) Kidney (iv) brain (v) live, and (vi) spleen of 5XFAD mouse.


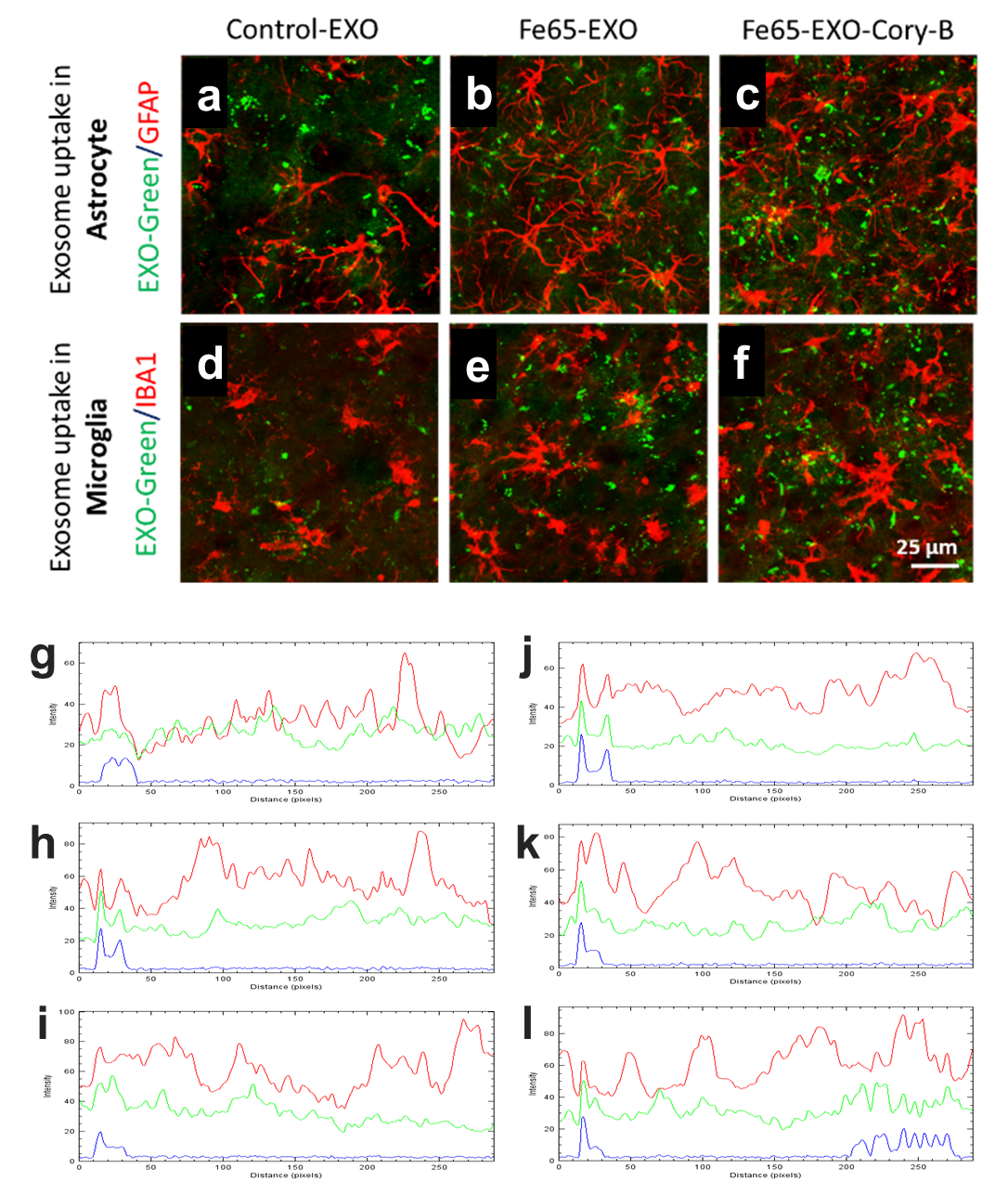


**Figure. S6.** **Evaluation of exosomal uptake in astrocyte and microglia in the brain of AD mice. (a-c)** Representative immunofluorescence staining of GFAP (for astrocyte) in the hippocampus region of brain tissue of 5xFAD mice model, injected with Exo-Green labeled- (a) Control-EXO, (b) Fe65-EXO, or (c) Fe65-EXO-Cory-B. **(d-f)** Representative immunofluorescence staining of IBA1 (for microglia) in the hippocampus region of brain tissue of 5xFAD mice model, injected with EXO-Green labeled- (d) Control-EXO, (e) Fe65-EXO, or (f) Fe65-EXO-Cory-B. The scale bar in a-f: 25 µm. **(g-l)** The corresponding fluorescence intensity of (g-i) EXO-Green and GFAP, and (j-l) EXO-Green and IBA1, depicting the strength of their co-localization.


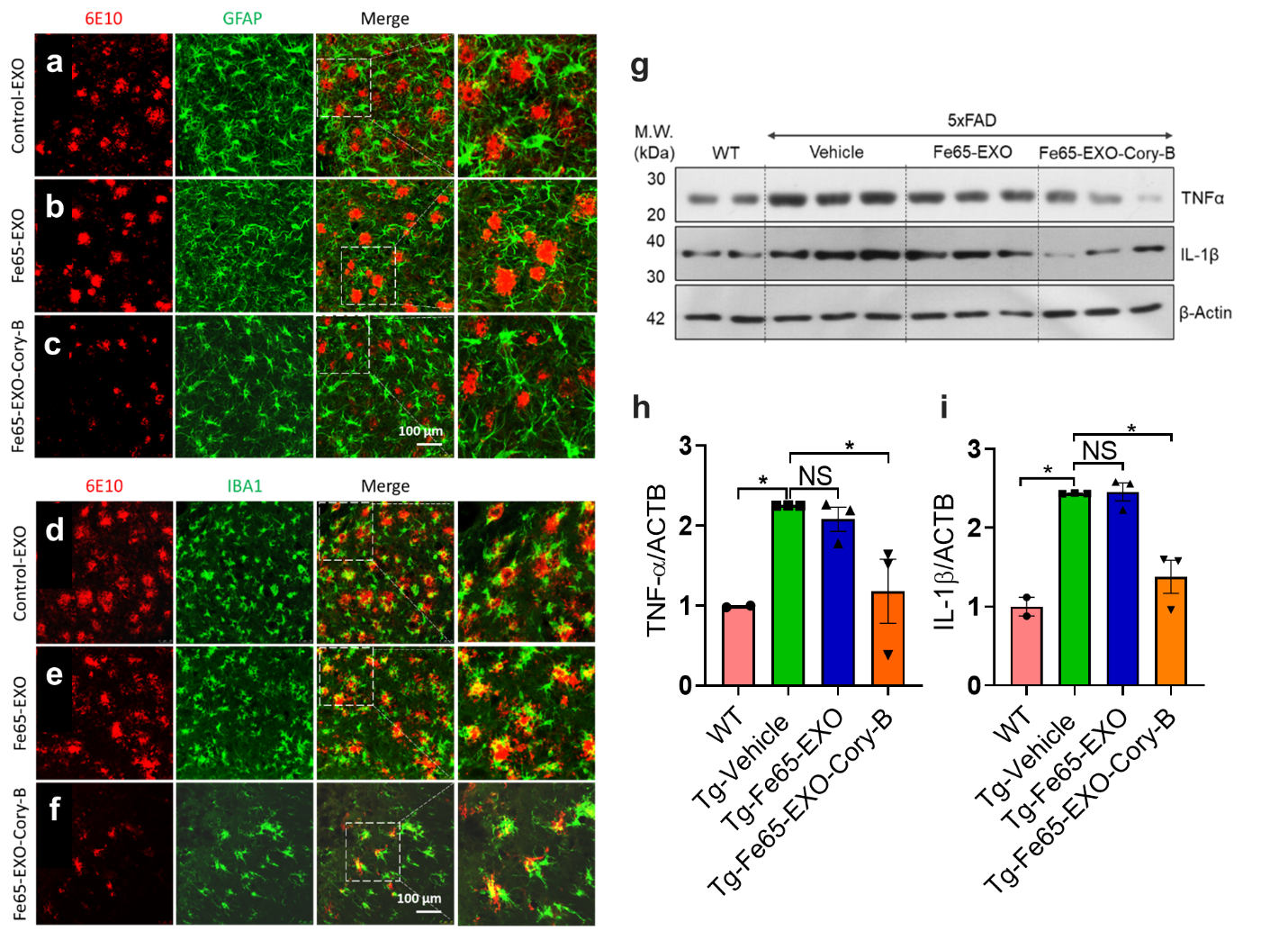


**Figure. S7.** **Evaluation of the effect of Fe65-EXO-Cory-B on neuroinflammation in the brain of AD mice. (a-c)** Representative immunofluorescence co-staining of amyloid beta using anti-6E10 antibody and astrocyte using anti-GFAP antibody in the hippocampus region of brain tissue of 5xFAD mice model, injected with (a) Control-EXO, (b) Fe65-EXO, or (c) Fe65-EXO-Cory-B. **(d-f)** Representative immunofluorescence co-staining of amyloid beta using anti-6E10 antibody and microglia using anti-IBA1 antibody in the hippocampus region of brain tissue of 5xFAD mice model, injected with (d) Control-EXO, (e) Fe65-EXO, or (f) Fe65-EXO-Cory-B. The scale bar in a-f: 100 µm. **(g-i)** Representative (g) western blot image and (h, i) the quantitative bar graphs, showing the expression levels of pro-inflammatory factors (TNFɑ and IL-1β) in the protein samples from the brain of WT mice and 5xFAD mice treated with vehicle, Fe65-EXO, and Fe65-EXO-Cory-B.


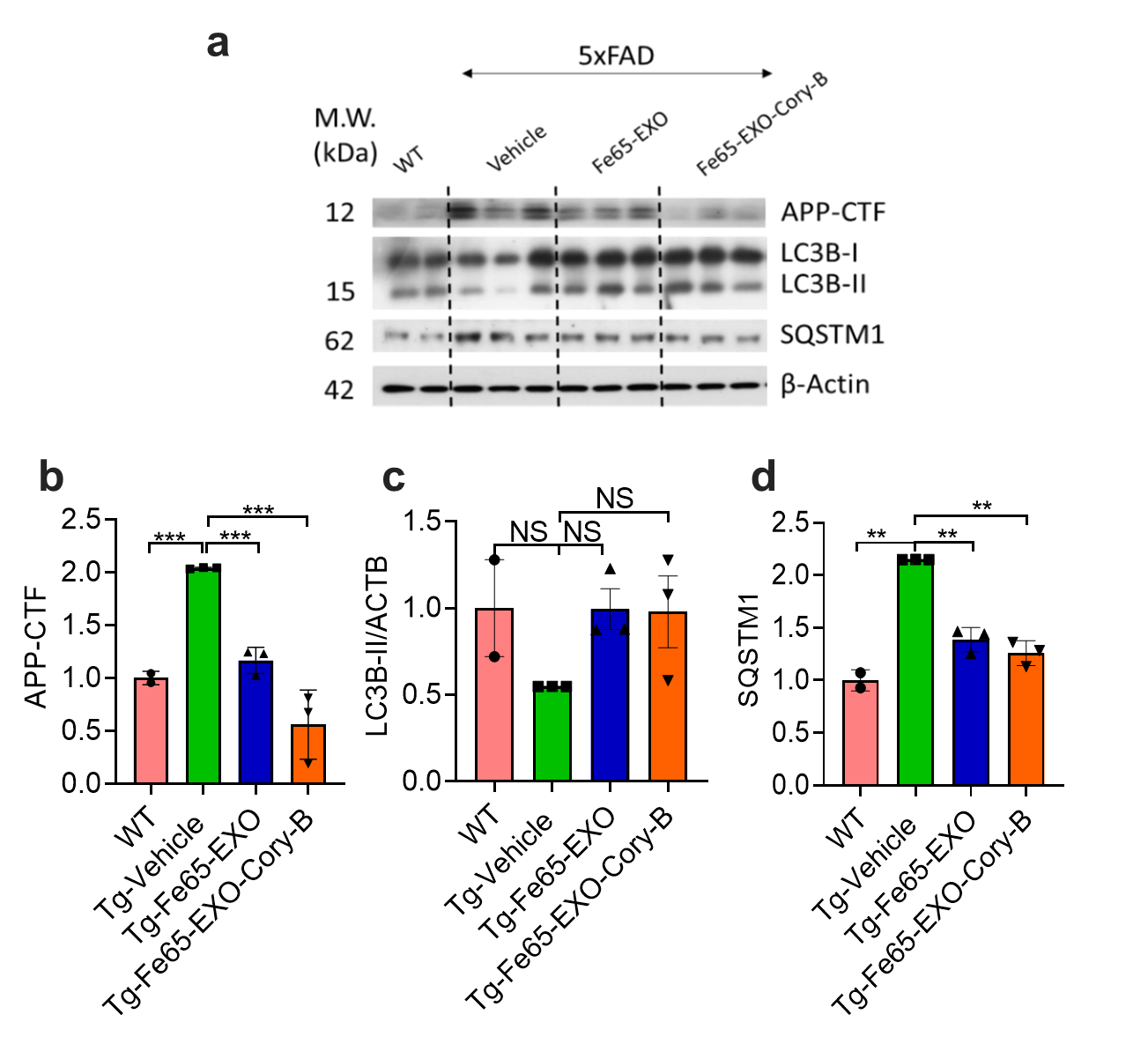


**Figure. S8. Evaluation of the effect of Fe65-EXO-Cory-B on the APP and autophagy in the brain of AD mice. (a)** Representative immunoblot showing the expression level of APP-CTF, and autophagy marker protein (LC3B-II, SQSTM1) in the brain samples of WT, and 5xFAD mice model, injected with vehicle (control), Fe65-EXO, and Fe65-EXO-Cory-B. **(b-d)** Quantitative bar graphs showing the expression of (b) APP-CTF, (c) LC3B-II, and (d) SQSTM1.


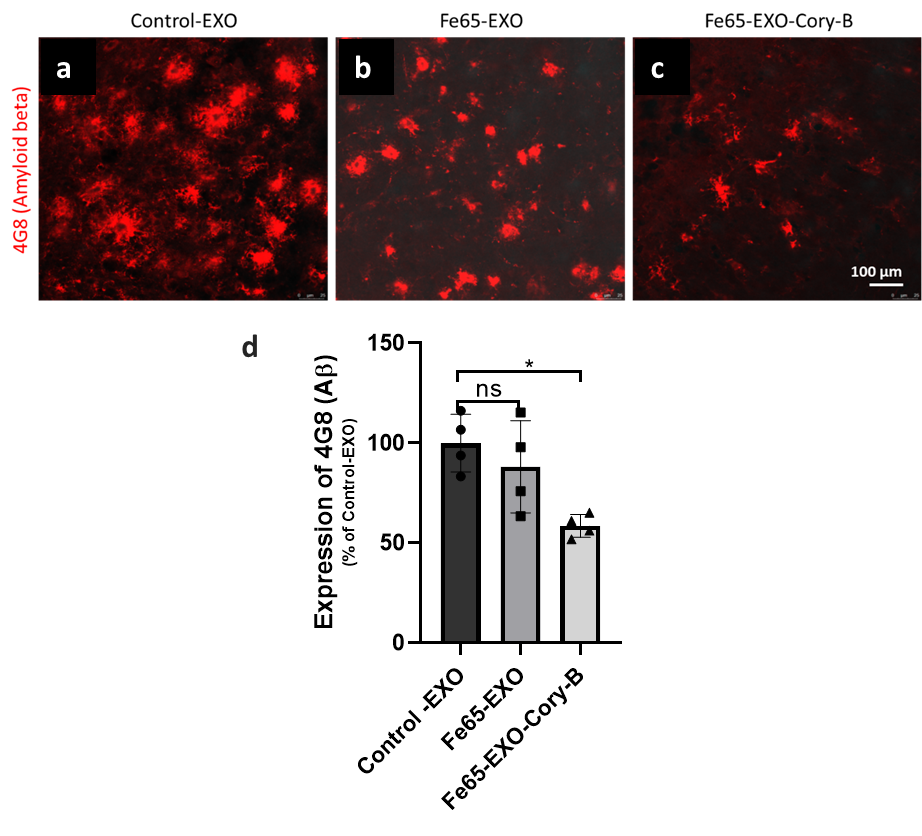


**Figure. S9. Evaluation of the effect of Fe65-EXO-Cory-B on the amyloid beta, and neuroinflammation in the brain of AD mice. (a-c)** Representative immunofluorescence staining of amyloid beta using anti-4G8 antibody in the hippocampus region of brain tissue of 5xFAD mice model, injected with (a) Control-EXO, (b) Fe65-EXO, or (c) Fe65-EXO-Cory-B. **(d)** The quantitative bar graph showing the expression of 4G8 for the amyloid beta in the hippocampus region of brain tissue of 5xFAD mice model, injected with Control-EXO, Fe65-EXO, or Fe65-EXO-Cory-B.


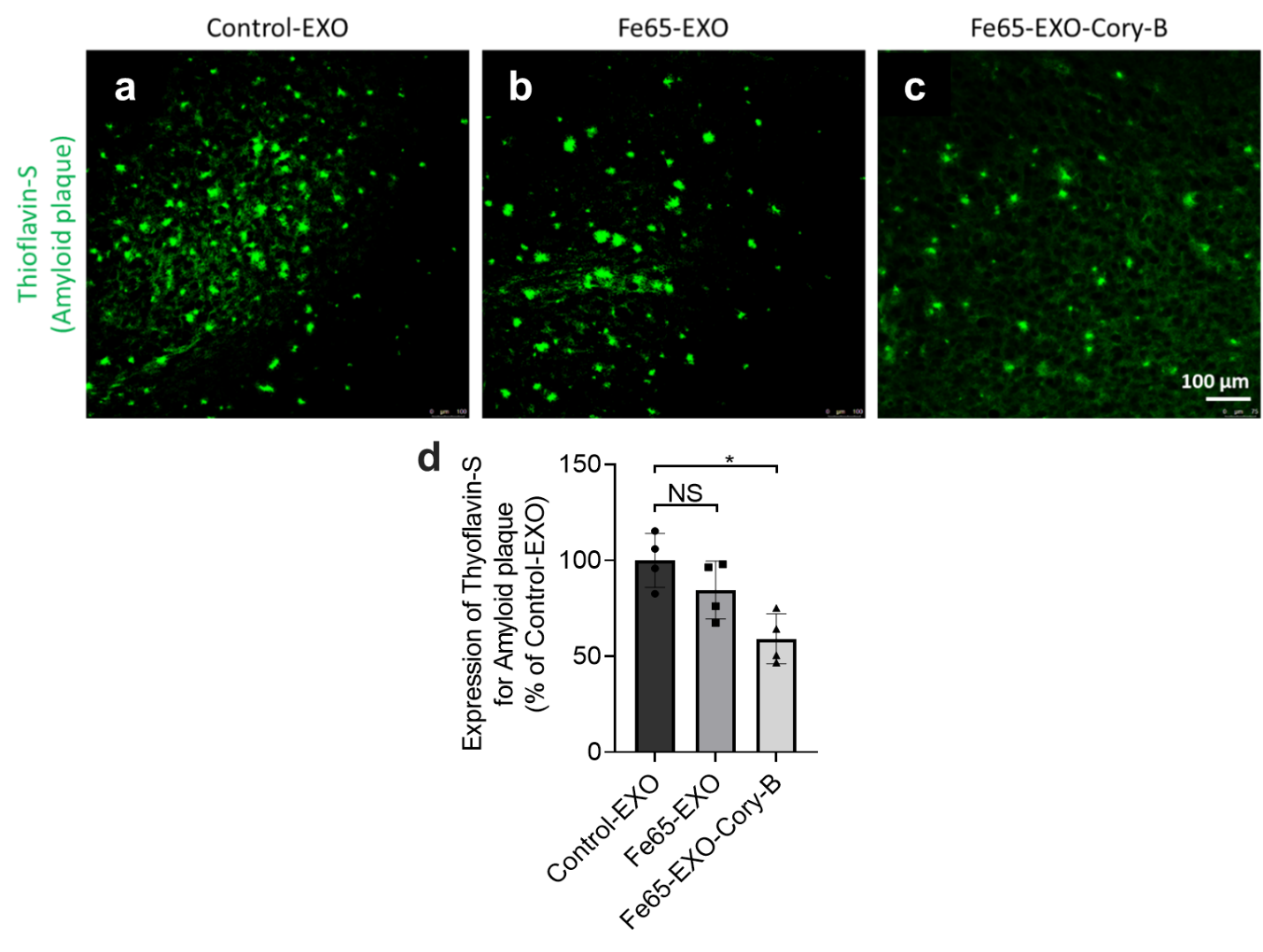


**Figure. S10. Evaluation of the effect of Fe65-EXO-Cory-B on the amyloid plaque in the brain of AD mice. (a-c)** Representative immunofluorescence staining of Thioflavin-S (THS) dye in the hippocampus region of brain tissue of 5xFAD mice model, injected with (a) Control-EXO, (b) Fe65-EXO, or (c) Fe65-EXO-Cory-B. **(d)** The quantitative bar graph showing the expression of Thyoflavin-S for the amyloid plaque in the hippocampus region of brain tissue of 5xFAD mice model, injected with Control-EXO, Fe65-EXO, or Fe65-EXO-Cory-B.


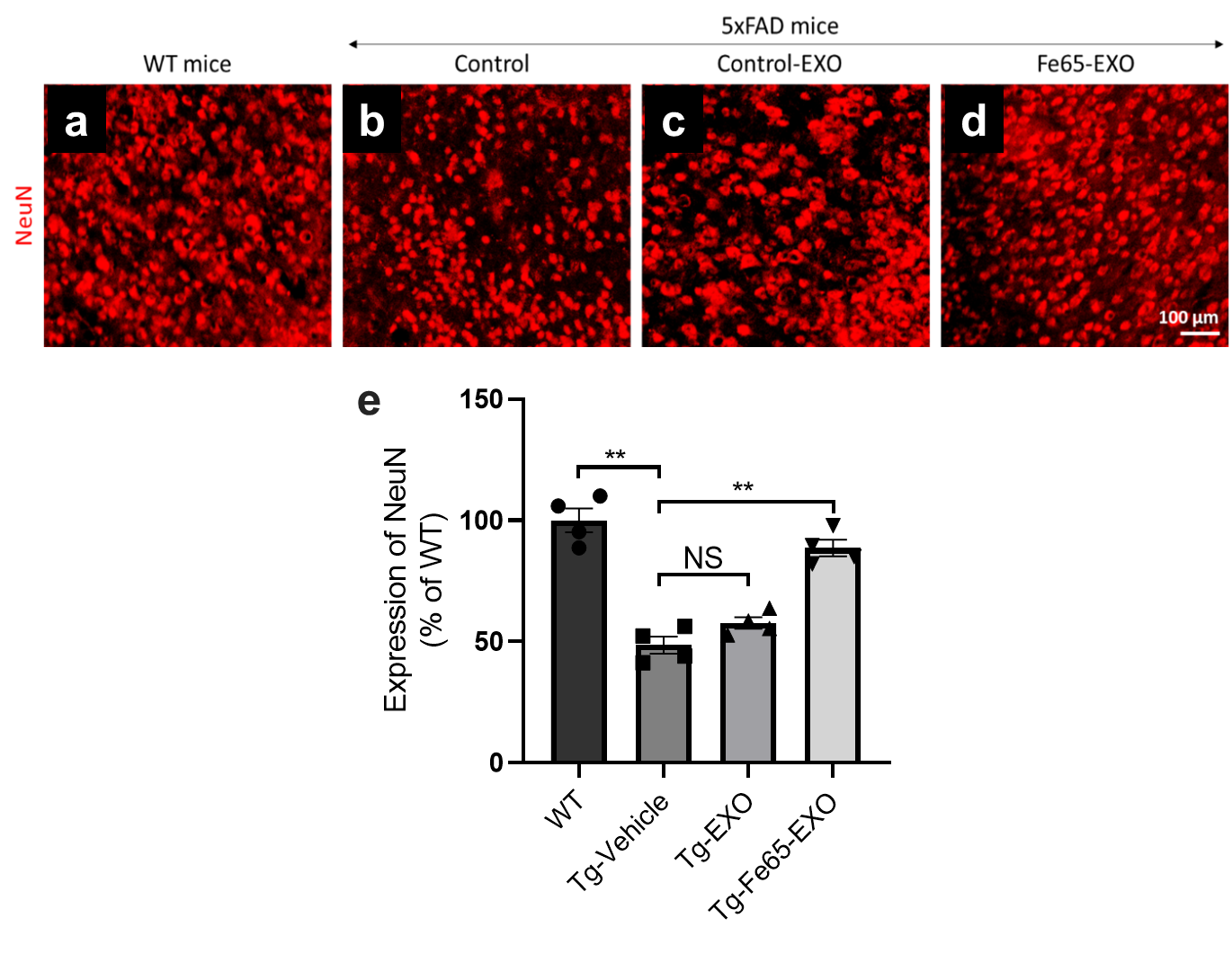


**Figure. S11. Evaluation of the effect of Fe65-EXO on the neurogenesis in the brain of AD mice. (a-d)** Representative immunofluorescence staining of NeuN in the hippocampus region of brain tissue of (a) WT, and (b-d) 5xFAD mice model, injected with (b) vehicle (control), (c) Control-EXO, (d) Fe65-EXO. **(e)** The quantitative bar graph showing the expression of NeuN as a marker for neurogenesis in the hippocampus region of brain tissue of WT, and 5xFAD mice model, injected with vehicle (control), Control-EXO, Fe65-EXO.


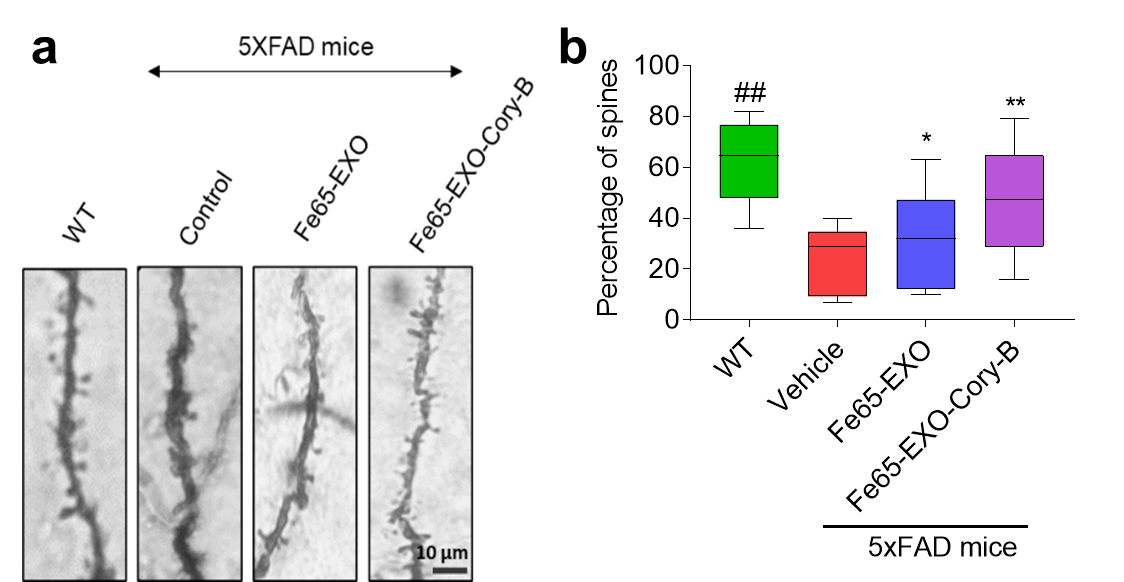


**Figure. S12. Evaluation of neuroprotective ability of Fe65-EXO-Cory-B in the AD mice. (a)** Representative images and **(b)** the quantitative bar graph, showing the Golgi staining of brain slice from the WT mice, and 5XFAD mice treated with vehicle, Fe65-EXO, and Fe65-EXO-Cory-B.


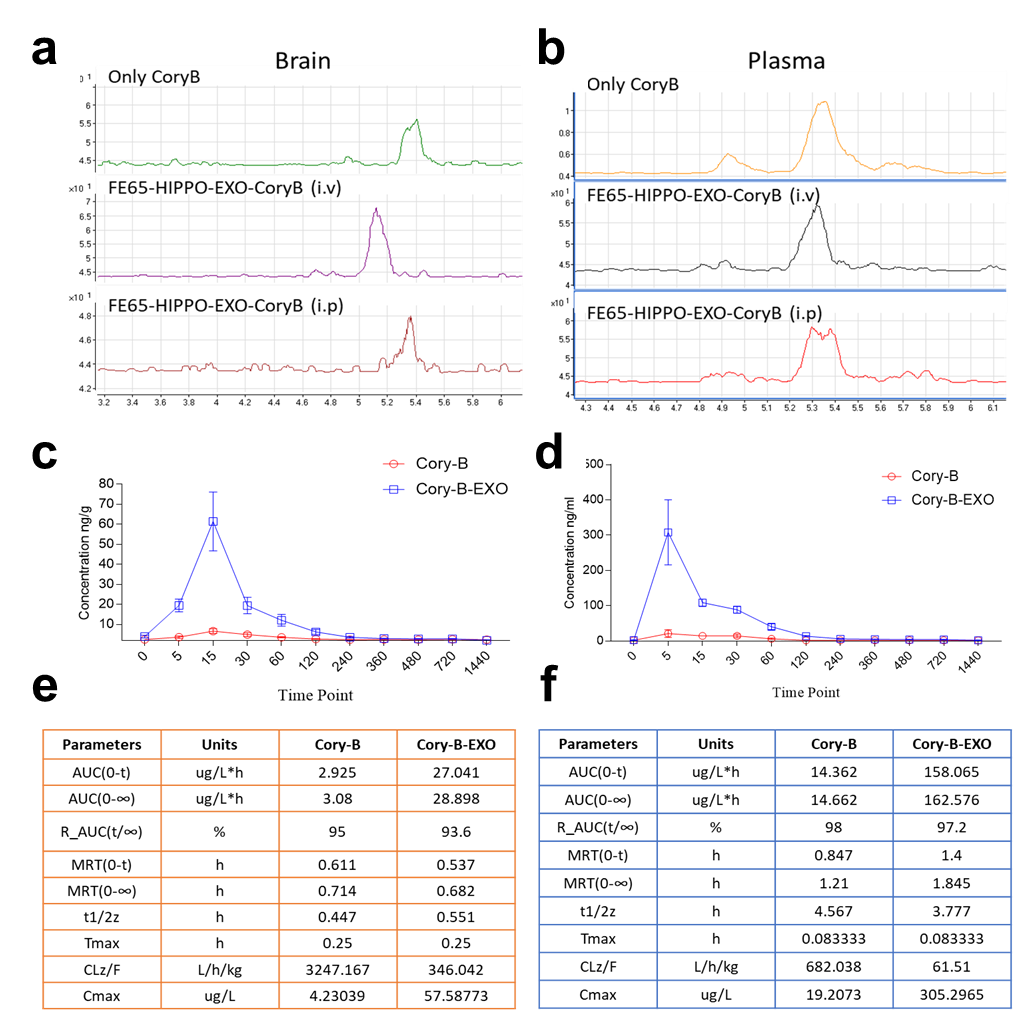


**Figure. S13. Bioavailability and pharmacokinetic study of exosomes carrying Cory-B. (a, b)** Representative LS-MS curves for the Cory-B alone, Fe65-EXO-Cory-B (i.v.), and Fe65-EXO-Cory-B (i.p.) in the biofluids of (a) brain and (b) plasma. **(c-f)** Representative time-dependent concentration showing (c, d) area under curve for the Cory-B and Fe65-EXO-Cory-B in the (c) brain and (d) plasma, and (e, f) the corresponding pharmacokinetic parameters.


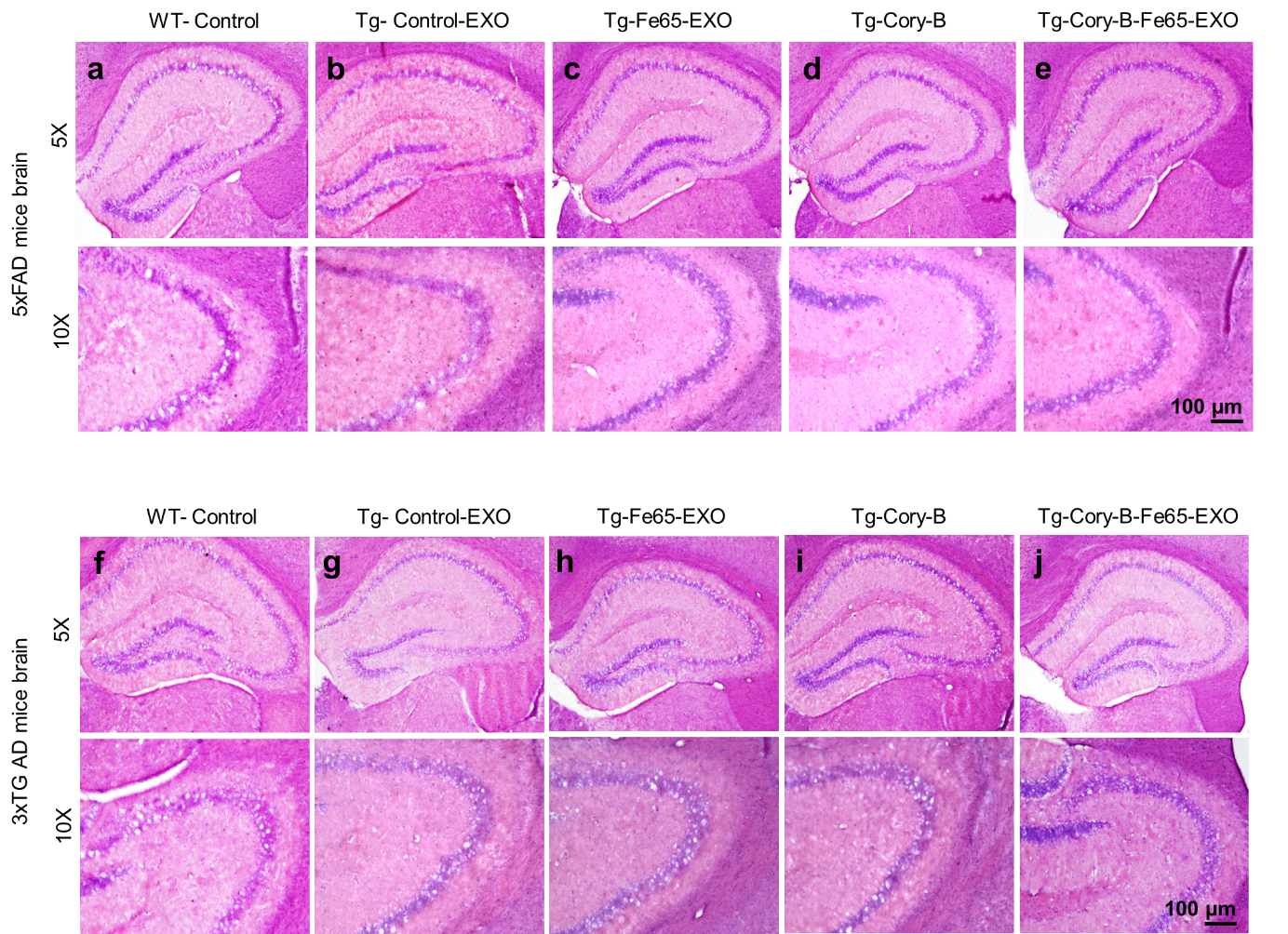


**Figure. S14.** **Safety evaluation of Fe65-EXO in 5XFAD and 3xTg-AD mice brain tissue.** Histological staining of the hippocampus region of brain of **(a-e)** 5XFAD, and **(f-j)** 3xTg-AD mice. The mice were divided into groups treated with Control-EXO, Fe65-EXO, Cory-B, and Fe65-EXO-Cory-B. (Scale bar = 100 μm).


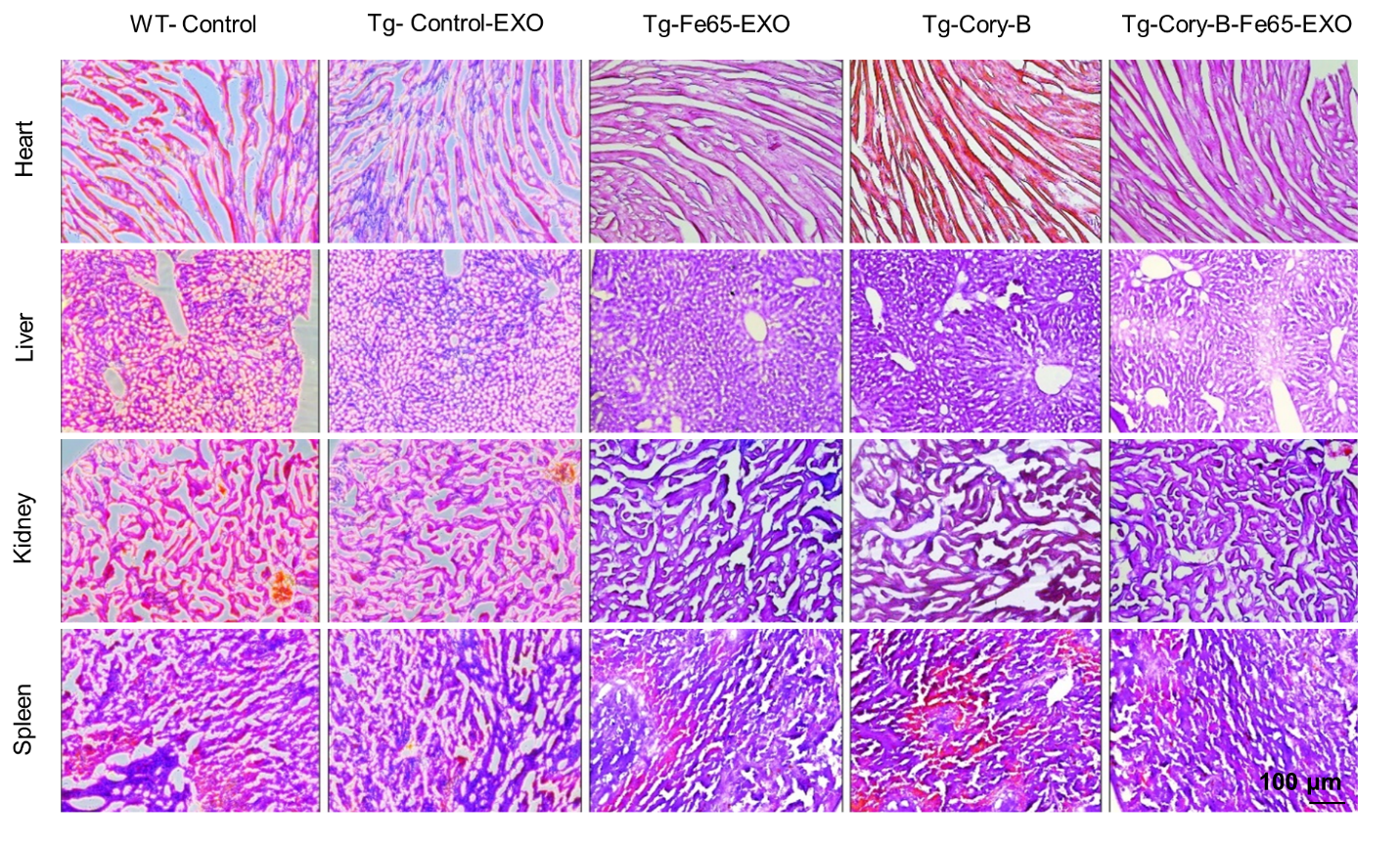


**Figure. S15. Safety evaluation of Fe65-EXO in different organs of 5xFAD mice.** Histological staining of tissue from heart, liver, kidney, and spleen of 5XFAD mice, injected with various groups viz. Control-EXO, Fe65-EXO, Cory-B, and Fe65-EXO-Cory-B. (Scale bar = 100 μm).


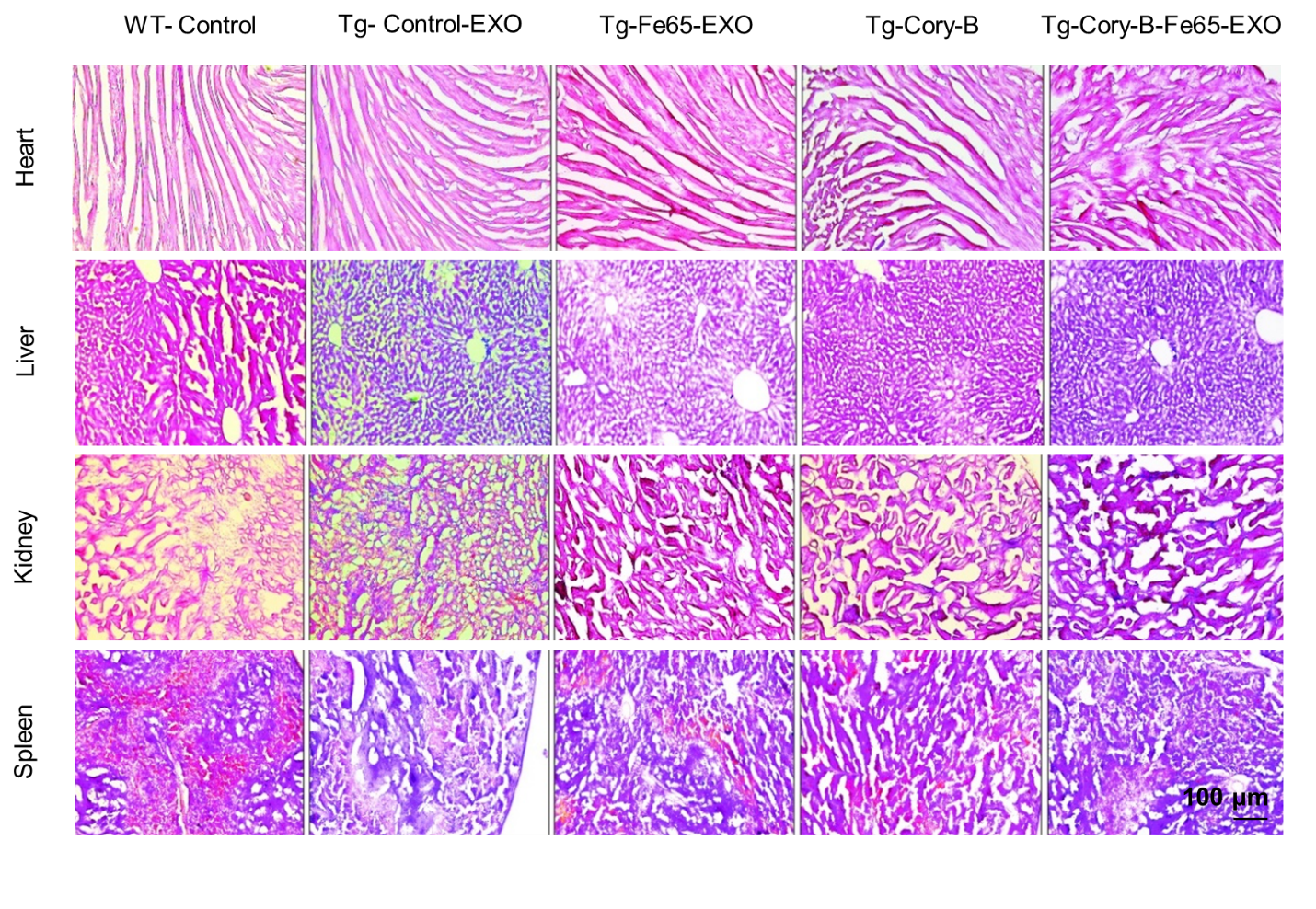


**Figure. S16. Safety evaluation of Fe65-EXO in different organs of 3xTg-AD mice.** Histological staining of tissue from heart, liver, kidney, and spleen of 3xTg-AD mice, injected with various groups viz. Control-EXO, Fe65-EXO, Cory-B, and Fe65-EXO-Cory-B. (Scale bar = 100 μm).


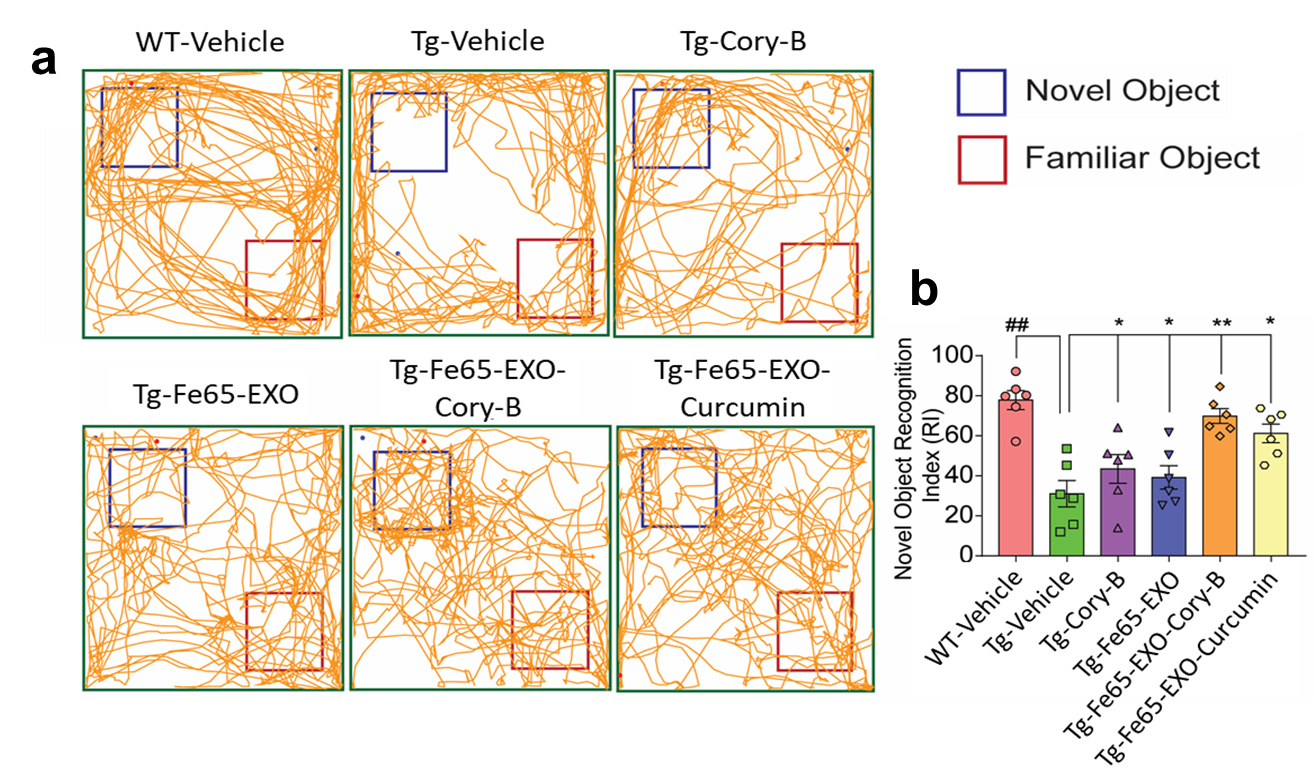


**Figure. S17. Evaluation of the effect of Fe65-EXO-Cory-B on the memory and learning ability in AD mice. (a)** Representative track records of the wild type mice, and 5xFAD mice model, injected with vehicle (control), Cory-B, Fe65-EXO, Fe65-EXO-Cory-B, and Fe65-EXO-Curcumin, using novel object recognition test set-up. **(b)** Quantitative bar graphs showing the change in novel object recognition index of 5xFAD mice model compared to the WT mice, and the assessment of object recognition index of 5xFAD mice injected with Cory-B, Fe65-EXO, Fe65-EXO-Cory-B, and Fe65-EXO-Curcumin compared to vehicle treated 5xFAD mice model. N= 6 mice/group. Data are represented as mean ± SEM. Comparison was done with student’s t-test: WT-Vehicle vs. Tg-Vehicle; Significance level: ^#^P<0.05, ^##^P<0.01, ^###^P<0.001. Comparison was done with one-way ANOVA with Dunnett’s multiple comparison test: Tg-Vehicle vs. Tg-Cory-B, Tg-Vehicle vs. Tg-Fe65-EXO, Tg-Vehicle vs. Tg-Fe65-EXO-Cory-B, Tg-Vehicle vs. Tg-Fe65-EXO-Curcumin; ^*^P<0.05, ^**^P<0.01, ^***^P<0.001.


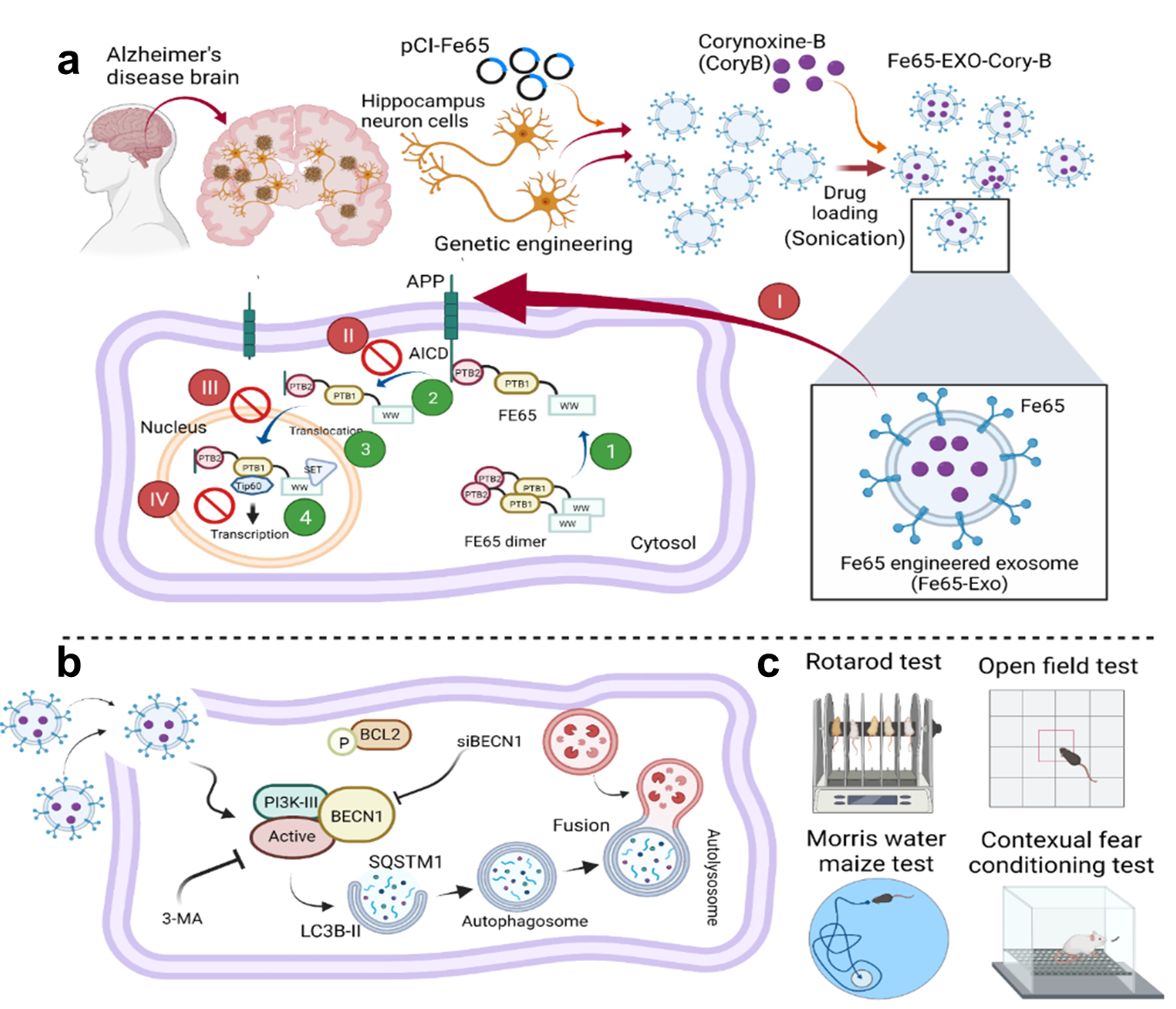


**Figure. S18. Fe65 engineered exosomes for APP targeted delivery of Cory-B in the brain of AD murine model. (a)** Representative schematic diagram showing APP targeted delivery of Cory-B loaded in Fe65 engineered hippocampus neuron cells-derived exosomes. In general, Fe65-mediated APP signaling involves a cascade of processes. First, a closed dimer of Fe65 is formed in the cytosol. Second, the dimer opens via binding to the APP mediated by the recruitment of phosphatidyl-inositol-4,5-bisphosphate (PIP_2_), leading to the formation of APP intracellular domain (AICD)/Fe65-PTB2 interaction. Third, after the cleavage of APP, the translocation of AICD-Fe65 complex to the nucleus takes place. Eventually, the process of transcription activation is initiated. These entire steps contribute towards the AD progression. However, the Fe65 expressed on the engineered exosomes can target and bind to the AICD via PTB2 domain of FE65 protein, which could hijack via blocking the binding of normally expressed FE65 with APP, and release Cory-B at the target site in the AD brain (based on the available known information). **(b)** Potential role of Fe65-EXO encapsulating Cory-B (Fe65-EXO-Cory-B) in the induction of autophagy in neuron cells via BECN1 or ATG5, or ATG7, leading to **(c)** the improvement of cognitive and locomotor behavior for ameliorating pathogenesis in murine AD mouse, as detected via rotarod, open field, Morris water maize, and contextual fear conditioning test.

**Table S1.** Specifications of antibodies used in the present study.

| **Antibody name** | **Source / Company** | **Catalogue number** | **Assays and dilution** |
| --- | --- | --- | --- |
| Anti-APP antibody | Thermo Scientific,  Waltham, MA, USA | 14-9749-82 | Western blotting  (WB) 1:1000 |
| Anti-ACTB/β-actin antibody | Santa Cruz, Dallas,  TX, USA | sc-47778 | WB, 1:10000 |
| Anti- SQSTM1/p62 antibody | Sigma-Aldrich, St. Louis, Missouri, USA | P0067 | WB, 1:20000 |
| Anti-LC3B antibody | Novus Biologicals, Littleton, Colorado, USA | NB100-2220 | WB, 1:20000 |
| Anti-ATG7 antibody | Cell Signaling Technology, Danvers, Massachusetts, USA | 8558S | WB, 1:1000 |
| Anti-BECN1 antibody | Santa Cruz, Dallas,  TX, USA | sc-10086 | WB, 1:1000 |
| Beta Amyloid-APP antibody | Invitrogen, Waltham, MA, USA | 51-2700 | WB, 1:1000 |
| Anti-FE65 antibody | Abcam, Cambridge, United Kingdom | ab5668 | WB, 1:1000 |
| Anti-CD63 antibody | Abcam, Cambridge, United Kingdom | ab216130 | WB, 1:1000 |
| Anti-4G8 antibody | Biolegend, United Kingdom | 800705 | IHC, 1:500 |
| Anti-NeuN antibody | Merck Millipore, Sigma Aldrich, USA | MAB377B | IHC, 1:1000 |
| Anti-IBA1 antibody | Abcam, Cambridge, United Kingdom | ab178846 | IHC, 1:1000 |
| Anti-GFAP antibody | Dako, Agilent, CA, USA | Z0334 | IHC, 1:1000 |
| Anti-TNFɑ antibody | Invitrogen, Waltham, MA, USA | MM350D | IHC, 1:1000 |
|  |  |  |  |
| Anti-IL-1β antibody | Abcam, Cambridge, United Kingdom | Ab254360 | IHC, 1:1000 |
